# Transcriptome changes induced by arbuscular mycorrhizal fungi in sunflower (*Helianthus annuus L*.) roots

**DOI:** 10.1038/s41598-017-18445-0

**Published:** 2018-01-08

**Authors:** Alberto Vangelisti, Lucia Natali, Rodolfo Bernardi, Cristiana Sbrana, Alessandra Turrini, Keywan Hassani-Pak, David Hughes, Andrea Cavallini, Manuela Giovannetti, Tommaso Giordani

**Affiliations:** 1 Department of Agriculture, Food, and Environment, University of Pisa, Via del Borghetto 80, I-56124 Pisa, Italy; 2 CNR, Institute of Agricultural Biology and Biotechnology UOS Pisa, Pisa, Italy; 3 Rothamsted Research, Harpenden, Hertfordshire, AL5 2JQ UK

## Abstract

Arbuscular mycorrhizal (AM) fungi are essential elements of soil fertility, plant nutrition and productivity, facilitating soil mineral nutrient uptake. *Helianthus annuus* is a non-model, widely cultivated species. Here we used an RNA-seq approach for evaluating gene expression variation at early and late stages of mycorrhizal establishment in sunflower roots colonized by the arbuscular fungus *Rhizoglomus irregulare*. mRNA was isolated from roots of plantlets at 4 and 16 days after inoculation with the fungus. cDNA libraries were built and sequenced with Illumina technology. Differential expression analysis was performed between control and inoculated plants. Overall 726 differentially expressed genes (DEGs) between inoculated and control plants were retrieved. The number of up-regulated DEGs greatly exceeded the number of down-regulated DEGs and this difference increased in later stages of colonization. Several DEGs were specifically involved in known mycorrhizal processes, such as membrane transport, cell wall shaping, and other. We also found previously unidentified mycorrhizal-induced transcripts. The most important DEGs were carefully described in order to hypothesize their roles in AM symbiosis. Our data add a valuable contribution for deciphering biological processes related to beneficial fungi and plant symbiosis, adding an *Asteraceae*, non-model species for future comparative functional genomics studies.

## Introduction

Arbuscular mycorrhizal (AM) symbioses are mutualistic associations between plant roots and soilborne fungi, involving about 80% of land plants. The fungal symbionts are represented by arbuscular mycorrhizal fungi (AMF; phylum Glomeromycota), a key group of beneficial obligately biotrophic microorganisms, which enhance plant growth and nutrition, receiving in exchange plant carbon compounds^1^. AMF increase the uptake and transfer from the soil to the host plant of mineral nutrients, in particular phosphorus (P), by means of large networks of extraradical hyphae, spreading from mycorrhizal roots to the surrounding environment^2,3^. Moreover, they provide a number of ecosystem services, improving plant tolerance to biotic and abiotic stresses and reducing the use of chemicals in agriculture^4^. Beyond improving plant nutrition and health, AMF induce changes in plant physiology, affecting the activity of host cell basic metabolism, *i.e*. plastid biosynthetic pathways and Krebs cycle^5,6^ and secondary metabolism, *i.e*. antioxidant enzymatic systems activity, isoprenoids, polyketides, and polyphenols biosynthesis^7,8^.

Studies aimed at analysing wide-scale gene reprogramming during the establishment and development of arbuscular mycorrhizal symbioses provided information on the molecular changes occurring in different tissues of a few plant species, such as *Medicago truncatula*, *Lotus japonicus*, *Pisum sativum*, tomato, potato, rice and soybean^9–15^. Large variations in gene expression were detected, depending on fungal symbiont identity, plant tissue and specific root cell/fungal structure location^9,13,16,17^. Different species of AM fungal symbionts were able to activate both shared and differential gene expression in their host plants^16–20^. Most work analysed mycorrhizal root transcriptomes, while only two studies detected up/down regulated genes both in shoot and root tissues, which varied with the host plant tested^9,17^. The analyses of gene expression in roots, where AM symbiosis is established, revealed changes both in plant and fungal transcriptomes, linked to mycorrhizal establishment and development^21–25^. Moreover, differential expression of some genes putatively involved in the accommodation of fungal symbionts in root cells during appressorium formation and in arbusculated cells was detected by laser-microdissection^13,23,26,27^. Several studies focused on root gene expression at different stages of mycorrhizal symbiosis establishment, showing differential activation and modulation of gene expression levels^11,13,16,21,28–31^.

Most of the data reported so far on transcriptome profiles of mycorrhizal plants were obtained by using microarrays or macroarrays, as well as suppression subtractive hybridization and differential display^21,27,32^. Such studies provided useful information on mycorrhizal regulated transcripts, although limited to the annotated genes available for the different host plant species and tissues. Recently, the RNA-seq technology was used to detect whole transcripts in mycorrhizal and non-mycorrhizal *Solanum lycopersicum* fruits and leaves^33,34^ and *Oryza sativa* and *L. japonicus* roots^10,14^. The use of such a technique showed that important gene functional classes, including post-translational regulation, signalling, transport, biotic and abiotic stresses and hormone metabolism, were differentially regulated in leaves of mycorrhizal tomato plants, compared with non-mycorrhizal ones^34^. The other work on tomato carried out using RNA-seq showed an up-regulation of genes related to photosynthesis, stress response, transport, amino acid synthesis and carbohydrate metabolism functions, and a down-regulation of genes belonging to cell wall, metabolism and ethylene response pathways gene ontology (GO) classes in fruits produced by mycorrhizal plants, compared with non-mycorrhizal ones^33^. Handa *et al*. (2015) found 3,641 differentially expressed genes, mostly up-regulated, during arbuscular mycorrhizal development in *L. japonicus* roots and belonging to transport and secretion functional classes, whereas Fiorilli *et al*. (2015) detected 1,035 up-regulated genes in *O. sativa* mycorrhizal lateral roots compared with non-mycorrhizal ones.

*Helianthus annuus* belongs to the largest Angiosperm family, the *Asteraceae*. It is widely cultivated, one of the four most important sources of vegetable oil. Native to North America, it was domesticated by native Americans, before being introduced in Europe and bred to become a globally important crop^35^. It is generally accepted that even in North America the most widespread cultivars are derived from materials reintroduced from Russia after breeding^36^, indicating a strong reduction of genetic variability in the cultivars, compared with the wild genotypes, that are adapted to a very large range of environments^37^. The sunflower genome is large (about 3.3 Gbp) and has recently been sequenced^38^. It is largely (around 80%) made of repetitive elements^39–41^ with high genetic polymorphism, especially among wild and domesticated genotypes^42^. Gene expression in sunflower has been studied in different conditions, for example in response to biotic and abiotic stresses, such as oxidative-stress, temperature, wounding, and pathogen infection^43–45^. However no data are available on the interaction, in terms of gene induction and/or repression, between sunflower and AMF. Recently, a study on mycorrhizal root colonization in sunflower wild accessions, cultivars, and inbred lines, reported that wild accessions were more susceptible to colonization than cultivars^46^. Such relationship between susceptibility to AMF and degree of domestication was also reported by other studies^47,48^ and suggested to be driven by soil characteristics combined with differences in root traits.

Here, for the first time, we used RNA-seq technology in order to assess the expression of differentially regulated genes at early and late stages of mycorrhizal symbiosis establishment in the cultivated sunflower.

## Results

### AM colonization of sunflower roots

**Figure 1:**
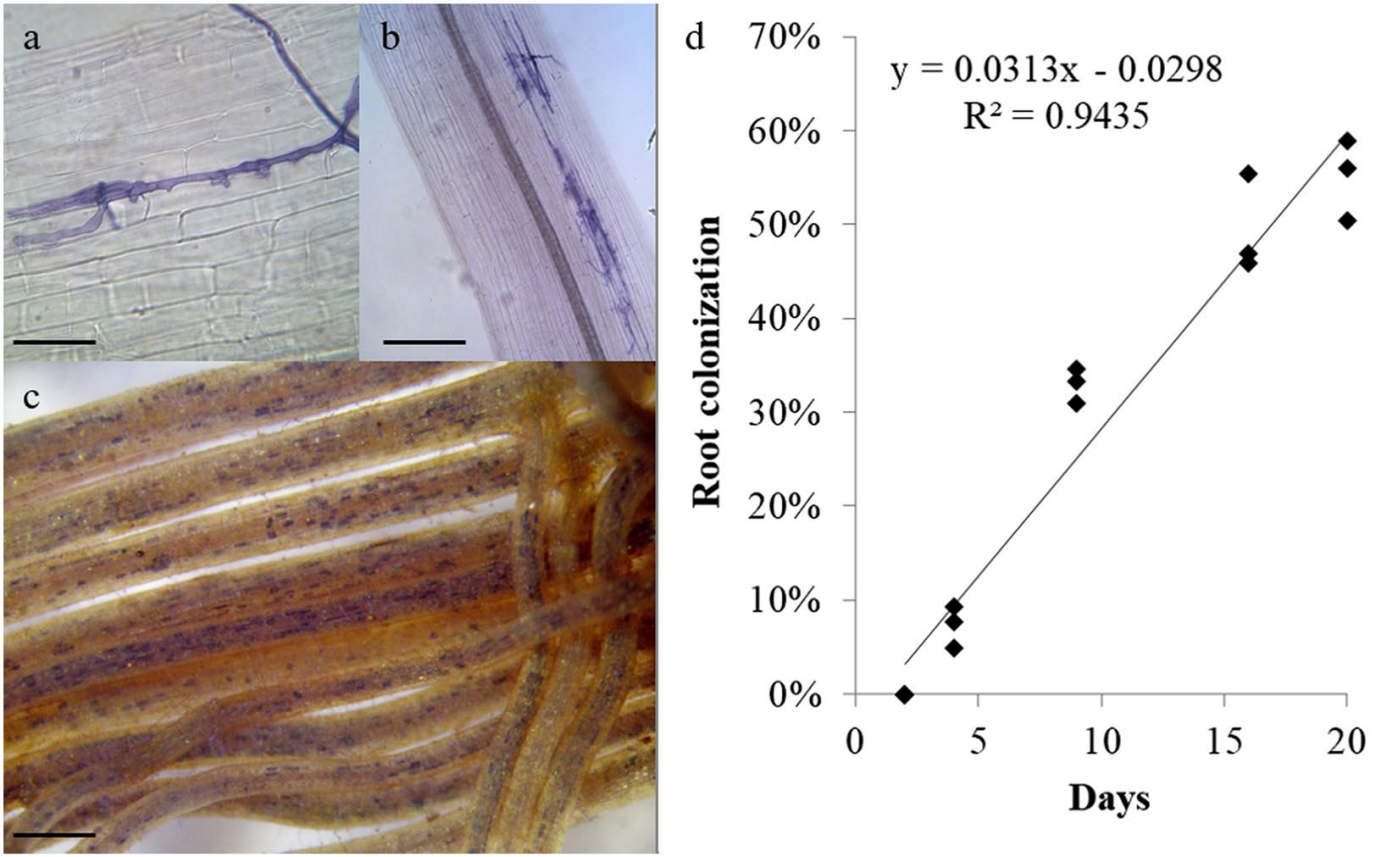
Micrographs showing trypan blue (**a**,**b**) and succinate dehydrogenase localization and trypan blue staining (**c**) of *Helianthus annuus* roots during colonization by the arbuscular mycorrhizal fungus *Rhizoglomus irregulare*. (**a**) Appressorium developing a penetration peg on *H. annuus* roots after 4 days of culture; scale bar = 30 µm; (**b**) fungal entry points formed by *R. irregulare* after 4 days of culture; scale bar = 80 µm; (**c**) *H*. *annuus* roots after 16 days of culture, showing extensive mycorrhizal colonization; scale bar = 3 mm; (**d**) time course of *H. annuus* mycorrhizal colonization across the different experimental time points. R^2^ represents the regression coefficient, y indicates the linear equation (n = 3).

*R. irregulare*-inoculated roots collected after 2 days of culture did not show any fungal structure, whereas two days later 7.3 ± 1.3% of root length was colonized. At this stage, mycorrhizal colonization was mainly represented by entry points, about 80 ± 21 per root system (0.3 ± 0.06 entry points cm root-1), although only 28% of them developed arbuscules (Fig. 1a,b). Mycorrhizal colonization increased at successive harvests, reaching 49.4 ± 3.0% and 55.1 ± 2.5% after 16 and 20 days of culture, respectively (Fig. 1c,d). After 16 days of culture, entry points raised to 1,230.7 ± 78.4 per root system (4.4 ± 0.4 entry points cm root-1) and 98% of them developed arbuscules.

### cDNA sequencing and aligning on reference predicted transcriptome

Twelve high quality cDNA libraries were prepared using RNA isolated from control and *R. irregulare* inoculated plants (Table 1).

**Tabelle 1:** Summary statistics for the Illumina sequencing and mapping against *Helianthus annuus* reference transcriptome HanXRQ.

Library nr. (and treatment)	Number of raw reads	Number of reads after trimming	Number of mapped reads on HanXRQ transcriptome	% of mapped reads on HanXRQ transcriptome
1 (C4)	48,052,338	45,262,022	42,298,895	93.45
2 (C4)	22,150,504	22,017,463	20,335,682	92.36
3 (C4)	18,724,363	18,464,157	17,356,730	94.00
4 (C16)	20,021,732	19,825,159	18,765,939	94.66
5 (C16)	55,479,243	54,193,826	50,403,351	93.01
6 (C16)	60,628,585	59,400,530	54,577,580	91.88
7 (M4)	52,342,074	50,361,161	46,184,688	91.71
8 (M4)	18,198,073	17,911,868	16,717,830	93.33
9 (M4)	19,798,867	18,640,381	17,195,413	92.25
10 (M16)	40,633,983	40,186,929	32,182,748	80.08
11 (M16)	21,494,687	21,349,112	17,098,190	80.09
12 (M16)	63,492,221	62,865,831	54,835,563	87.23

C4 = uninoculated roots after 4 days of culture, C16 = uninoculated roots after 16 days of culture, M4 = mycorrhizal roots after 4 days of culture with *Rhizoglomus irregulare*, M16 = mycorrhizal roots after 16 days of culture with *Rhizoglomus irregulare*.

Illumina sequencing generated 441,016,670 sequence reads, each 100 nt in length, encompassing about 29 GigaByte of sequence data. The total number of reads per library ranged from 18.72 to 63.49 million (Table 1). After removal of ambiguous and low-quality reads, 430,478,439 reads, 85 nt in length were retained (Table 1).

Reads from the 12 libraries were aligned to the reference transcriptome of *Helianthus annuus* ^38^. The percentage of mapping reads of each sample to the reference predicted transcriptome ranged from 80.08% to 94.66% (Table 1).

### Analysis of differentially expressed genes

We studied the expression of 52,243 predicted transcripts included in the *Helianthus annuus* reference transcriptome^38^. Analysis was limited to genes with RPKM > 1 in at least one of the three replicates of each treatment. By this method, we selected 33,332 expressed genes.

**Figure 2:**
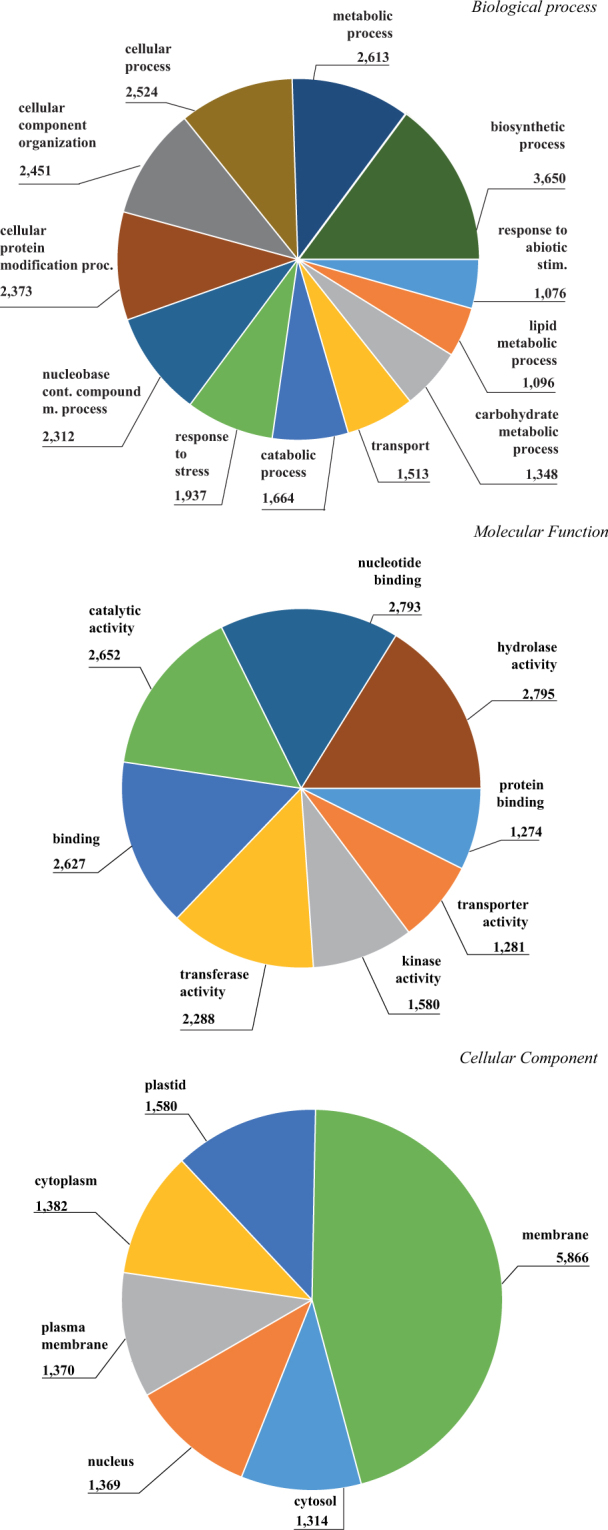
GO slim pie distribution of expressed transcriptome of *Helianthus annuus* roots, inoculated with *Rhizoglomus irregulare*. Cut off was set on 1,000 transcripts per GO term.

GO terms analysis performed on the gene expressed in mycorrhizal roots showed 3,650 and 2,613 genes belonging to “biosynthetic process” and “metabolic process” within the macro-functional class Biological Process; “hydrolase activity” (2,795 transcripts) and “nucleotide binding” (2,793 transcripts) were the most abundant terms in Molecular Function class; top GO terms among Cellular Component ontologies were “membrane” (5,866 genes), and “plastid” (1,580 genes) (Fig. 2).

Overall, 726 differentially expressed genes (DEGs) were detected in sunflower roots inoculated with the AM fungus *R. irregulare*, compared with uninoculated controls. This relatively low number of DEGs is comparable to those found in other plants inoculated with *R. irregulare* ^10,14^.

### Differentially expressed genes a**t the early mycorrhizal stage**

**Figure 3:**
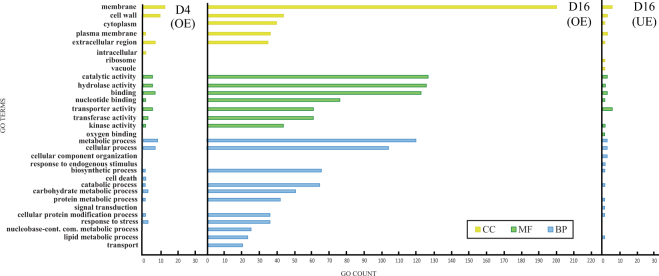
GO slim distribution of differentially over and under expressed genes of *Helianthus annuus* roots during early and late stage of symbiosis (4 days and 16 days after inoculation with *Rhizoglomus irregulare*). CC = Cellular Component, MF = Molecular Function, BP = Biological Processes.

Comparing *H. annuus* control with mycorrhizal roots 4 days after inoculation (D4), we detected only 19 significantly over-expressed genes. No significant under-expressed transcripts were found in D4 versus control plants. Within biological processes, the most abundant GO-terms of D4 differentially expressed genes were “metabolic process” and “cellular process”; among molecular functions, the most frequent terms were “binding”, “hydrolase activity” and “transporter activity”; cellular component class showed “membrane” and “cell wall” as major terms (Fig. 3).

Amongst differentially expressed genes of D4 plants we identified several transcripts encoding stress response proteins such as germin-like proteins and chitinase, and genes encoding polypeptides with protease activity (Subtilisin-like and carboxypeptidases). Other DEGs included those encoding a nrt1 protein of the ptr family, two UDP-glycosyltransferases, a lysM domain receptor-like kinase 3, an inorganic phosphate transporter, and a glutathione s-transferase (Supplementary Table 1).

### Differentially expressed genes at the late mycorrhizal stage

At the second harvesting time point, 16 days after sunflower root inoculation with the AMF *R. irregulare* (D16), the comparison between control and mycorrhizal roots showed 694 over- and 13 under-expressed genes, respectively (Supplementary Fig. 1).

The greatly increased number of DEGs in D16, compared with D4, may be ascribed to the increasing level of AM root colonization. Furthermore, the number of over-expressed DEGs in D16 plants was considerably higher than that of under-expressed DEGs (Supplementary Fig. 1).

GO terms analysis on D16 DEGs showed that “catalytic activity” and “hydrolase activity” ontologies were the most frequent in the Molecular Function class; the most represented Biological Process terms were “metabolic process” and “cellular processes”; “membrane” and “cell wall” were the two top terms in Cellular Component class (Fig. 3).

Some under-expressed genes were found in mycorrhizal D16 roots, although fewer than the over-expressed transcripts of the same root sample. Regarding Biological Processes, the most represented term was “metabolic process” (2 genes); within Cellular Components the top term was “membrane”; “transporter activity” was the most frequent term in the Molecular Function class (Fig. 3).

**Figure 4:**
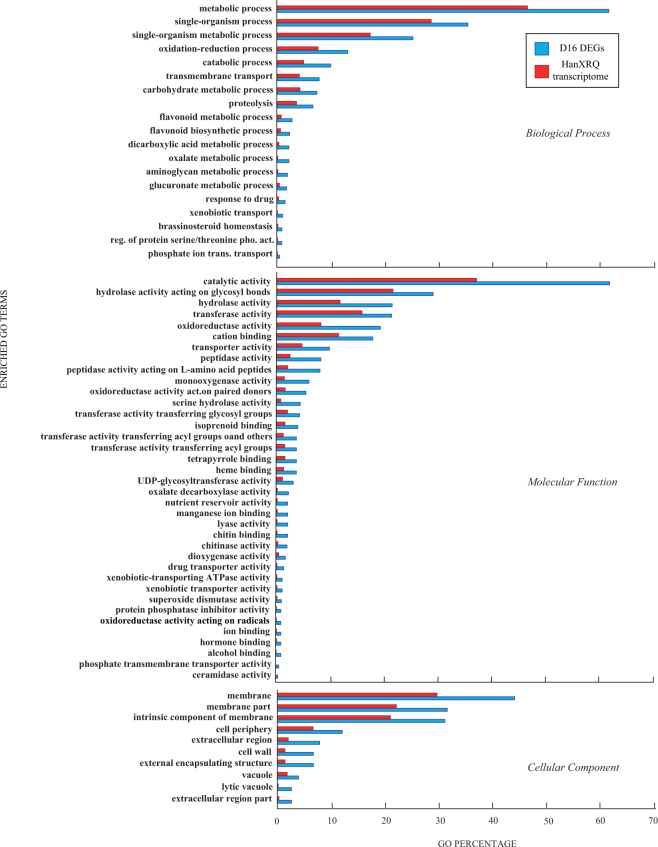
Enriched GO term distribution for differentially over-expressed genes of *Helianthus annuus* roots 16 days after inoculation with *Rhizoglomus irregulare*. Only summarized enriched GO terms were shown. Abscissa shows percentage of GO terms per D16 DEGs (blue bar) compared to GO terms of whole reference predicted transcriptome (red bar).

Gene ontology enrichment analysis showed that only the group of over-expressed genes in D16 was significantly enriched (P < 0.05). Using REVIGO, “hydrolases”, “oxidoreductase” and “catalytic activity” categories were enriched in the late stage of colonization: “catalytic activity” were the most enriched among Molecular Functions, and “metabolic process” among Biological Processes (Fig. 4). The most enriched term in the Cellular Component class was “membrane” (Fig. 4).

Among those over-expressed genes in D16 roots with high fold changes (logFC > 11) we retrieved genes encoding putative transcription factors (an ap2-like ethylene-responsive transcription factor, a DELLA protein, a PHR1), transporter proteins (clathrin assembly protein, an ammonium transporter 3, an ascorbate-specific transmembrane electron transporter, two GDSL esterase lipases) oxidation-reduction activity proteins (two cytochrome p450, several vacuolar-processing enzymes, a putative chitinase 2, several beta-amyrin 28-oxidases, a reticuline oxidase, two SKU5s) and other proteins involved in signal trasduction, cell functioning and metabolism (a vinorine synthase, proteins containing ankyrin repeats, an abscisic acid 8 -hydroxylase 3) (Supplementary Table 1).

In addition, among other over-expressed transcripts at D16 with logFC < 11 were those encoding for proteins involved in stress-defense responses (heat shock proteins, several hydroxyproline-rich glycoprotein, peroxidases, and several lectins) and putative proteins of root remodeling and growth (an acidic endochitinase, two auxin response factors and alpha-tubulins, CDPK, and several ABC transporters) (Supplementary Table 1).

Amongst under-expressed genes of *H. annuus* mycorrhizal roots at D16 we detected many transcripts encoding for SWEET-like bidirectional sugar transporters, one transcript belonging to a pathogen-related protein and a peroxidase gene. (Supplementary Table 1).

Noteworthy nineteen DEGs were found to be shared among mycorrhizal roots of *H. annuus* after 4 and 16 days of culture with *R. irregulare*. All shared transcripts showed an increasing log fold change between the first and the second time point (Table 2).

**Tabelle 2:** Differentially expressed genes in *H. annuus* roots after 4 (D4) and 16 days of culture (D16) with the arbuscular mycorrhizal fungus *R. irregulare* (log FC: log_2_ fold change).

Functional class	Genes identifier	D4 logFC	D16 logFC	Transcript description
Catalytic activity (response to stress)	HanXRQChr09g0257681	4.32	7.57	germin 2 type 1
	HanXRQChr10g0295541	3.74	6.41	germin 2 type 1
	HanXRQChr10g0295581	4.25	7.16	germin 2 type 1
	HanXRQChr03g0087411	7.07	11.76	Mavicyanin
	HanXRQChr03g0087431	8.46	13.28	Mavicyanin
	HanXRQChr05g0159761	5.01	10.97	chitinase type 2
	HanXRQChr12g0354981	7.77	14.36	Rhicadhesin receptor
Hydrolase activity (protein metabolic process)	HanXRQChr01g0024241	6.57	10.61	Subtilisin-like protease
	HanXRQChr03g0072271	6.06	9.32	serine carboxypeptidase II type 3
	HanXRQChr13g0419211	4.04	11.20	subtilisin-like protease
	HanXRQChr03g0087471	5.32	10.87	Subtilisin-like protease SDD1
Kinase activity and signal transdcution	HanXRQChr06g0174451	4.76	7.98	lysM domain receptor kinase type 3
Transferase activity	HanXRQChr11g0350641	5.63	12.45	UDP-glycosyltransferase
	HanXRQChr15g0488781	5.10	10.79	UDP-glycosyltransferase
Transporter activity	HanXRQChr15g0472261	7.31	11.40	NRT1 PTR
	HanXRQChr04g0127841	4.88	11.18	ammonium transporter 3 member type 1
	HanXRQChr10g0280991	4.32	11.05	inorganic phosphate transporter 1 type 4 isoform × 2
	HanXRQChr15g0490031	5.42	10.10	Nodulin-26-like
No class	HanXRQChr03g0074111	3.99	11.30	Predicted protein

### qRT-PCR validation analysis

In order to validate RNA-seq data, the expression of five genes selected among those differentially expressed between M4 and M16 samples were analysed by qRT-PCR experiments. As shown in Supplementary Figure 2, all genes were differentially expressed, confirming the accuracy of transcriptome profiling obtained with RNA-seq.

## Discussion

A large number of terrestrial plant species establish symbioses with AMF, leading to morphological and functional changes in roots. Next generation sequencing techniques can be conveniently used for analysis of transcriptome profiling during plant/fungus interactions. In this work, RNA-Seq experiments in roots of sunflower after *R. irregulare* inoculation gave a comprehensive view of changes in gene expression, confirming the activation of peculiar AMF regulated genes in sunflower and identifying new genes so far not associated with the establishment of AMF symbioses. Here, we detected a total of 19 and 694 genes over-expressed in mycorrhizal roots at early and late stage of colonization, respectively. Number of DEG is lower to the one retrieved during AM symbiosis in *Lotus japonicus* and *Oryza sativa* ^10,14^. Differences may be related to slightly lower percentage of hyphae colonization in sunflower roots compared to these studies, to different experimental conditions used and to species specific responses.

Among new genes which were not described so far as involved in AMF symbiosis, we identified, for example, those encoding several BAHD-acyltransferases, an oxoglutarate dependent dioxygenase, and a stellacyanin-like protein (Supplementary Table 1).

Overall GO analysis of DEGs showed a number of ontologies: “biosynthetic process”, “hydrolase activity” and “membrane” were the most frequent functional classes. In particular, the GO term “membrane” reflects the major cell membrane rearrangement associated with fungal invasion, “biosynthethic process” suggests host transcriptomic, metabolic and transport activity alteration during symbiosis, and “hydrolase activity” the wide protein recycling associated to cell wall modification (Figs 3 and 4).

Global gene expression data show that D16 mycorrhizal roots were far more active in transcription than D4 root samples. Furthermore, we noticed an unbalanced number of over-expressed genes in comparison with under-expressed genes, as described for other mycorrhizal plant species, for example *Lotus japonicus* ^14,49^. Indeed, very few transcripts were significantly under-expressed in sunflower AM roots.

One of the major obstacles in studying AMF symbiosis is the difficulty in synchronizing the initial developmental events leading to the establishment of the symbiosis^26^. At the early stage time-point (D4), low AMF colonization, mainly represented by entry points with a few developed arbuscules, occurred in inoculated sunflower roots. Consequently, it can be suggested that DEGs in D4 roots could represent genes regulated in the early phases of fungal colonization. During the late phase of symbiosis establishment (D16), when half the length of host roots were colonized by *R. irregulare*, a higher number of DEGs were found compared to D4, reflecting the advanced stage of root colonization. DEGs were differentially expressed at both time-points, although they were modulated between D4 and D16. This finding is in accordance with the asynchronous nature of the mycorrhizal colonization process, as D16 samples showed fungal structures representative of both early and late stages of the symbiosis, as new entry points may be produced by extraradical hyphae, giving rise to secondary colonization^26^.

Differentially expressed genes of *H. annuus* roots in symbiosis with *R. irregulare*, shared between different time points, encompass genes of symbiosis development, maintenance and functioning and belong to a large number of GO terms. Many of these GO terms and specific genes will be discussed hereafter in relation to their modulation, expression and function.

Differentially over-expressed genes in D4 roots mainly belong to the functional classes “response to stress”, “cell wall” and “transporter activity”, which are largely represented during early interactions between plants and AMF^11,28^. Interestingly, all D4 DEGs were differentially expressed also in D16 plants, with increasing expression levels at the latter time point (Fig. 3). Indeed, among DEGs involved in plant defense, 3 and 15 transcripts of Germin-like protein were detected in D4 and D16 plants (Table 2) respectively, suggesting a modulation of this multi-gene family as AMF colonization proceeds. Similarly, the number of induced protease-encoding genes increased from 4 to 27 (including genes encoding subtilisins and carboxypeptidases) between the two time points, confirming the involvement of this family in maintaining cell functions and recycling cell wall proteins during mycorrhizal symbioses^14,50^.

A similar induction profile was observed also for genes involved in transporter activity, which increased from 4 to 60 between early and late stage of colonization (Fig. 3, Table 2), confirming the importance of transporter genes modulation during the symbiotic interaction^11^. Among these DEGs, those encoding an NRT1 PTR protein, involved in NO_3_- and auxin transport, an inorganic phosphate transporter and the phosphate and water transporter nodulin-26-like protein were confirmed to be expressed at both early and late stages of symbiosis establishment^11,51,52^.

A LysM-domain receptor kinase encoding gene is the only signal transduction gene identified amongst D4 DEGs. The number of DEGs of this family increased to 7 in D16 roots, including some leucin rich receptors (Table 2, Supplementary Table 1). LysM is required for the earliest cellular and physiological symbiotic responses and its expression was detected in *M. truncatula* roots 8 days post inoculation^28,53^.

UDP-glycosyltransferase is one of the stress-responsive gene belonging to the GO term “transferase activity” and was over-expressed even in D4 AM roots, increasing its logFC between D4 and D16 (Table 2). Its activity should combat oxidative stress and pathogen infection. Genes of this family were reported to be differentially expressed in AM plants^14,20,54^, but they were never previously described in association with the early steps of mycorrhizal establishment.

DEGs observed only at D16, we will discuss them in relation to their functional classes.

The GO term “response to stress” contains several genes encoding transcripts involved in plant stress and defense, and is one of the most represented among DEGs (Figs 3, 4). It has been established that common defense pathways are activated in both pathogenic and beneficial plant-fungal interactions, as a result of fungal elicitors recognised by the plant^28,53^.

New transcripts implied in fungus-plant relationship are BAHD-acyltransferases, which are here described for the first time in AM symbioses, and a transferase super-family including Vinorine synthases, which play a central role in the endogenous formation of specialized monoterpenoid secondary metabolites, functioning as defensive compounds upon pathogenic infections^55,56^. Accumulation of terpenoids belonging to phytoalexins was also detected in mycorrhizal roots of *Solanum tuberosum* and *Solanum lycopersicum* ^33,57^.

Another plant defense related gene, encoding Glutathione-S-transferase, is active in M16 sunflower roots. Its activation was already observed in *Medicago truncatula* ^58^.

Other over-expressed transcripts encode phytyltransferases; these genes play a role in the isoprenoid metabolic pathway. Usually, isoprenoids are highly expressed as secondary metabolites during AM formation, conferring resistance to stress with activation of plant defense system^59–61^.

“Catalytic activity” is another wide functional class involved in plant defense, cell wall loosening and oxidative stress defense. DEGs of this functional class encode for germin-like proteins, the monocopper oxidase SKU5 and several peroxidases^62,63^ (Supplementary Table 1), all known to be induced during AM symbiosis^13,29,49,64,65^.

Other DEGs encoding defense and stress-responsive proteins were found in mycorrhizal sunflower roots after 16 days of culture. These genes encode pathogen-related proteins, such as chitinases and endoglucanases, and several peroxidases, which were shown to be differentially expressed in other mycorrhizal plant species^66–69^.

Other defense-related putatively encoded lectins and enzymes involved in isoprenoid and terpenoid metabolism were reported to accumulate specifically in mycorrhizal roots^23^. In connection with isoprenoid and terpenoid metabolism, an over-expressed gene in D16 mycorrhizal roots encoded a beta-amyrin 28-oxidase that catalyzes the oxidation of beta-amyrin to oleanolic acid^70^.

Another new transcript involved in symbiosis events is an oxoglutarate dependent dioxygenase encoding gene. This gene, for the first time associated with AM symbiosis, was over-expressed in D16 mycorrhizal roots. Oxoglutarate dependent dioxygenases are involved in alkaloid production, whose content (hyoscyamine and scopolamine) was previously reported to increase in mycorrhizal roots of *Datura stramonium* ^71^.

It is interesting to note that some pathogen-related and disease-resistance proteins encoding genes were significantly down-regulated in sunflower AM roots 16 days after inoculation (Supplementary Table 1). Although a partial overlap between symbiotic and pathogenic signaling has been reported^72^, transcript levels of some of these genes were much lower in plant roots colonized by beneficial fungi than in those infected by root pathogens. This reduced expression was associated with a very restricted localization of transcripts in arbuscule containing cells^73^. The repression of transcription of some plant defence genes suggests that AMF are able also to trigger mechanisms in plant roots that suppress defence response allowing colonisation to proceed.

Reception of molecular signals and signal transduction are crucial biological events for symbiosis establishment. DEGs with high logFC related to signal formation and transduction encode P450 oxidases (Supplementary Table 1), which are known to be involved in the production of strigolactones and co-expressed both in the plant and in the fungus during AM symbiosis^14,74–76^.

Signals released by the fungus activate transcription factors in root cells. Among highly over-expressed putative transcription factors, we detected genes encoding a PHR1, several DELLA proteins involved in gibberellin signaling regulation in AM^77–80^ (Supplementary Table 1), and many ethylene responsive factors (ERFs). PHR1 is a transcription factor belonging to the MYB family that binds to promoters of most genes positively or negatively affected by Pi starvation^81,82^, hence it may be considered a candidate gene product involved in Pi transport regulation. Ethylene-responsive factors and the ERF Required for Nodulation (ERN) transcription factor are induced in response to AMF, and ERN-like proteins encoding genes showed specific expression in arbusculated cells^23^.

Some over-expressed transcripts are related to fatty acid metabolism, as for example the GDSL esterase/lipase. The role of these proteins is not fully known, but their remarkable expression during AM symbiosis, mainly in arbusculated cells, has been correlated with the production of signal molecules and membrane components involved in the synthesis of periarbuscular membranes^14^.

A transcript encoding a calcium-dependent protein kinase 1-like protein was over-expressed in sunflower D16 mycorrhizal roots, compared with controls (Supplementary Table 1). Calcium-dependent protein kinases (CPKs or CDPKs) are important Ca^2+^ sensors in signaling processes during the establishment of symbioses between host plants and both AMF and rhizobia^83,84^.

As reported above, previous studies suggested that hormonal balance of mycorrhizal roots is tightly linked to the development of fungal colonization and root structural changes. The expression pattern of genes putatively encoding for auxin and gibberellin biosynthesis showed that transcripts encoding GA oxidases and auxin response factor were over-expressed in D16 mycorrhizal roots (Supplementary Table 1).

Gibberellin content increased in the roots of mycorrhizal plants compared with uninoculated controls in parallel with the accumulation of transcripts involved in their biosynthesis^85–87^.

The role of auxins in mycorrhizal symbioses is not clear, as biochemical and molecular studies showed variable auxins and auxin-related molecules levels, although data on auxin mutants suggested that auxin perception is required for arbuscule development and mycorrhizal symbiosis establishment, at least by modulating strigolactone levels^79,88,89^.

A transcript homologous to zeatin O-glucosyltransferase differentially accumulated in D16 mycorrhizal roots (Supplementary Table 1). Such proteins were reported to be involved in cytokinin pathways with the role of catalyzing the formation of o-xylosylzeatin from zeatin^29^.

Many over-expressed transcripts putatively encoding transporter proteins were found in both D4 and D16 mycorrhizal roots, including an inorganic P transporter, a multidrug resistant ABC transporter, nitrate and ammonium transporters, and aquaporins (Supplementary Table 1). These genes are known to be highly expressed during mycorrhizal interactions and play a pivotal role in symbiotic signaling and nutrition pathways^90–93^.

ABC transporter genes are required for arbuscule development^94^ and were over-expressed in D16 mycorrhizal roots samples.

In this work, genes encoding inorganic P, ammonium and nitrate transporters and NIP class of aquaporins were over-expressed in D4 and D16 mycorrhizal roots, and a larger number of highly expressed isoforms was detected at the late stage of the symbiosis, confirming previous data^16,29,95,96^. The nitrate transporter Nrt1 was shown to play a pivotal role also in auxin transport and consequently in lateral root formation^52^.

Another newly identified transcript is related to an aquaporin-like encoding gene over-expressed in D16 AM roots that encodes for a stellacyanin-like protein, exhibiting sequence similarity with nodulin-like transporters^97^ (Supplementary Table 1). A gene encoding for a putative stellacyanin-like protein, CASLP1, was strongly expressed in *Capsicum annuum* plants after inoculation with different pathogenic bacteria and fungi, possibly due to water losses or to a role of this protein in limiting pathogen spread^98^. Although at a lower level of over-expression, a probable tonoplast intrinsic protein (TIP) aquaporin was also detected in D16 mycorrhizal roots.

A 9-fold over-expressed sequence recovered from D16 mycorrhizal roots showed homology to a bidirectional SWEET16-like sugar transporter (Supplementary Table 1), characterized as an exporter of sucrose and monosaccharides^99^, and could play a role in regulating the transfer of plant carbon resources at the plant–fungus interface. Potato SWEETs showed differential expression regulation in response to AMF, although further studies are needed to clarify SWEETs’ role in sugar partitioning during mycorrhizal colonization process^100^.

Plant cell walls form a dynamic extracellular matrix that controls growth and development and, at the same time, mediates most plant/fungal interactions. During AM symbiosis the cortical layers are reached by fungal hyphae branches that, after contact, lead to unique structures called arbuscules, and this process requires the activation of several genes^101^. Many transcripts involved in cell wall and membrane shaping, functional to hyphal penetration and arbuscule development, were highly expressed, such as that encoding SKU5 protein, which exhibits sequence similarity with multiple copper ascorbate oxidase and laccase^102^ (Supplementary Table 1). Other over-regulated transcripts encode carboxypeptidases, subtilisin-like proteases and a type 1 ATPase, all belonging to the GO term Hydrolase and involved in cell remodeling and membrane transport. Here a considerably higher logFC of the expression of some subtilisins was detected in D16 mycorrhizal roots, compared with D4. This result is similar to that reported in roots of *L. japonicus* inoculated with *R. intraradices* ^50^.

In D16 mycorrhizal roots, transcripts encoding proteins functioning in cell wall and membrane shaping were detected, such as a ripening-related protein, a Clathrin vesicles protein, some vacuolar processing enzymes and Ankyrin-repeat containing proteins, as previously reported^52,103,104^ (Supplementary Table 1).

EG45 domain is typical of the expansin family^105^, a superfamily of proteins that play a pivotal role in cell wall loosening, thus increasing root cell wall plasticity. Expression of these proteins is triggered by fungal contact or by a diffusible fungal factor and is a prerequisite to accommodate the fungus in plant root cells^27,106^. Expansin-encoding genes showed up-regulation in mycorrhizal roots of *M. truncatula* and *C. sativus*, and their products were detected both in the interface matrix and in mycorrhizal root cell walls^28,107^. A gene encoding a protein similar to *Prunus armeniaca* major allergen, over-expressed in D16 mycorrhizal roots, showed a high similarity to a β-expansin^108,109^ (Supplementary Table 1).

Many D16 over-expressed genes belong to the GO term “carbohydrate metabolic process” (Fig. 2). Genes included in this GO, such as those encoding endoglucanases, galacturonases, hydroxyproline-rich glycoproteins, cellulose synthase and ascorbate oxidase, are mainly related to root cell wall degradation and remodeling and were expressed in root cells containing fungal structures^101,110,111^.

Tubulin encoding genes were over-expressed in D16 mycorrhizal roots (Supplementary Table 1), confirming previous findings on tubulin upregulation in *S. lycopersicum* roots colonized by *R. intraradices*, compared with uncolonized roots^112^.

Plant-fungal symbiosis stimulates new adventitious roots contributing to lateral root growth as showed in symbioses of *Allium porrum* and *Prunus cerasifera* with *F. mosseae* and *R. intraradices* ^113,114^. Auxin response factors (ARF) are transcription factors involved in adventitious root formation and regulate auxin responsive genes. The auxin response factor 18-like transcript detected here (Supplementary Table 1) was not reported before in mycorrhizal plants, whereas other transcripts encoding putative auxin response factors were induced during colonization of *O. sativa* and *M. truncatula* by *R. irregulare* ^18,65^. Such an expression pattern might be related, as in other ARFs, to lateral root formation and growth^115^.

## Conclusions

In this work, a comprehensive transcriptome analysis of roots of sunflower in symbiosis with the AM fungal symbiont *R. irregulare* was carried out adding data from a plant species belonging to *Asteraceae*, the largest Angiosperm family, which was not investigated yet in this respect. Hence, this is a valuable contribution to deciphering gene expression related to plant symbiosis, for future comparative functional genomics analyses. Results confirmed the importance of AM-modulated transcription of genes involved in plant defence, signal transduction, hormonal balance, nutrient transport and host cell shape in the activation of mechanisms controlling AMF establishment and spread in plant roots.

The repertoire of genes regulated at both early and late stages of the symbiosis allowed us to confirm AMF-regulated genes as well as to identify new genes which were not previously described as involved in AMF symbiosis, for example those encoding a BAHD-acyltransferase, an oxoglutarate dependent dioxygenase, and a stellacyanin-like protein.

Such results can guide application research targeted at exploiting metabolic changes occurring in commercially-valuable crops when colonized by arbuscular mycorrhizal symbionts in field conditions, in pursuit of sustainable and resilient agricultural processes.

Further studies are in progress to elucidate gene expression in the development of AM symbiosis by the comparison of transcriptome profiling in cultivated and wild genotypes of sunflower, showing different susceptibility to AMF.

## Materials and Methods

### Plant material and treatments

Fourty seeds of *H. annuus* HA412-HO inbred line (USDA acc. number PI 642777) were germinated on moistened filter paper in Petri plates. The root system of one week old plantlets was individually placed between a 90-mm diameter membrane (cellulose acetate and cellulose nitrate mixture, 0.45 µm pore diameter size, MF-Millipore™) and a 100-cm^2^ nylon net (41 µm mesh, Millipore™). Spores, mycelium and finely cut colonised roots of *R. irregulare* (formerly *Rhizophagus irregularis*) IMA6, obtained from pot-culture soil after wet sieving through a 100-µm-mesh size sieve, were spread onto all the nylon net surface of 20 plantlets. Uninoculated control plantlets received 10 ml of a filtrate, obtained by sieving the same pot-culture soil through a 50 μm pore diameter sieve and through Whatman N. 1 paper, to ensure a common AM fungal-associated microflora. In both inoculated plants and uninoculated controls another 90-mm membrane was placed on the nylon net, and these “sandwich systems” were placed in sterile 150-mm Petri plates containing steam-sterilized quartz grit. After sealing with Parafilm M, the lower half of each plate was wrapped in aluminium foil and plants were maintained in a growth chamber at 24 °C. Plants were supplied weekly with 6 ml half strength Hoagland’s solution. The progression of mycorrhizal colonization was monitored across time-points 48 hours apart on 3 replicate plants, by localizing succinate dehydrogenase activity^116^ and by staining roots with Trypan blue^117^. Percentage of AM fungal root colonization was determined by the gridline intersect method^118^.

### RNA extraction

On the basis of root colonization data, RNA-seq analyses were carried out on roots collected from 3 replicate *H. annuus* mycorrhizal and control plantlets harvested after 4 and 16 days of culture.

Whole roots were ground in liquid nitrogen and total RNA was isolated using the Logemann procedure^119,120^. Purification from genomic DNA was performed by digestion with DNaseI (Roche). Finally, RNA was purified with phenol/chloroform and precipitated with standard procedures. RNA quality was evaluated by electrophoretic, spectrophotometric and bioanalyzer analysis using a Bioanalyzer 2100 (Agilent Technologies, Santa Clara, CA).

### RNA sequencing and mapping procedures

For high-throughput sequencing, mRNA deriving from the experiment was converted to cDNA and 12 cDNA libraries (three replicates of mycorrhizal and control root systems at two harvesting times) were constructed with TruSeq RNA Sample Prep Kit (Illumina), according to the manufacturer’s protocol (Illumina Inc., San Diego, CA, USA). Illumina HiSeq 2000 sequencing yielded single-read sequences with length of 100 bp. The quality of the reads was checked using FastQC (v. 0.11.3) and overall quality was improved by trimming the reads using Trimmomatic^121^, with following parameters: crop = 95, headcrop = 10, minlen = 85.

High quality RNA-Seq reads were mapped using the CLC genomics workbench (v. 9.5.3).

Traces of ribosomal RNA contamination were removed from all libraries by mapping against sunflower ribosomal sequences downloaded from the NCBI database repository. The parameters used for ribosomal filtering were: length fraction = 0.5 and similarity fraction = 0.8.

cDNA sequences were mapped to the reference transcriptome of *Helianthus annus* inbred line XRQ^38^ (https://www.heliagene.org/HanXRQ-SUNRISE/) using stringent parameters (length fraction = 0.8 and similarity fraction = 0.8) but retaining mismatch penalties = 2 and gap penalties = 3 as set by CLC default parameters. Sequence reads are deposited in the Sequence Repository Archive (SRA) under the ID SRP124596.

### Differential expression analysis

Gene expression level was calculated as reads per kilobase per million reads mapped (RPKM) as described in Mortazavi *et al*., (2008). Besides unique reads, reads that occurred up to ten times were also included in the mapping analysis, because this strategy correctly estimates the expression of paralogous genes^122^. Gene expression was filtered to ensure RPKM > 1 in at least one library.

Raw counts deriving from CLC aligner were analyzed with the R statistical package EdgeR^123^. A pairwise comparison test was performed between mycorrhizal (M) and control libraries (C) after 4 and 16 days of inoculation with fungus (C4 vs. M4 and C16 vs. M16). The resulting *P* values were corrected for the FDR^124^ and significant genes identified with an FDR corrected *P* value < 0.05. The fold changes (FC) between controls and treatments were considered significant when the expression value of a gene was at least two-fold higher or lower than the control, thus splitting transcripts in two groups: up-regulated and down-regulated.

### Transcriptome functional analysis

GO terms for differentially expressed annotated transcripts were retrieved using a Blast2GO file provided by HanXRQ portal (https://www.heliagene.org/HanXRQ-SUNRISE/).

GO enrichment analysis with Fisher’s exact test on differential expressed genes versus whole predicted transcriptome was performed with Blast2GO analysis tools^125^ using FDR corrected P value < 0.05. Overall enriched GO terms were summarized using REVIGO with allowed similarity of 0.4^126^.

GO slim (Blast2GO tool) was performed in order to reduce the complexity of GO terms for functional analysis of differentially expressed transcripts.

### qRT-PCR analysis

We used qRT-PCR to validate RNA-seq expression profiles of five genes selected from those differentially expressed between M4 and M16 samples. One gene encoding E3 ubiquitin-ligase BRE1-like 1 (genome ID HanXRQChr05g0138451 and one gene encoding mitochondrial ubiquitin ligase activator of NFKB 1 (genome ID HanXRQChr10g0282971) were selected among genes showing stable expression profiles in RNA-seq analyses and used as internal reference genes in qRT-PCR analyses. Complementary DNA from 400 ng total purified RNA was prepared using iScript cDNA synthesis kit (BioRad, Hercules, CA, USA) according to the manufacturer’s protocol.

The synthesised cDNA was used for quantitative real-time polymerase chain reaction (qRT-PCR). Primers were designed using Primer3 software (Applied Biosystems) and are reported in Supplementary Table 2. qRT-PCR reactions (20 μL) were carried out with 10 ng of cDNA, 250 nM primers and 1X Fast SYBR Green Master Mix (Applied Biosystems, Foster City, CA, USA) following the manufacturer’s instructions. PCR was run in a StepOne realtime PCR System (Applied Biosystems, Foster City, CA, USA) using the recommended thermal cycling conditions (hold 95 °C, 20 s; 40 cycles 95 °C, 3 s; 60 °C, 30 s).

The qRT-PCR results were obtained from three biological replicates and three technical repeats for each treatment. The relative abundance of transcripts was calculated by using the 2−ΔΔCt method^127^. For statistical analysis a two tailed t-test was used.

### Accession codes

Sequence reads of transcriptome sequencing have been deposited in the NCBI sequence read archive under accession code SRP124596.

## Electronic supplementary material

Supplementary information
