# Supplementary material for: Transcriptome changes induced by arbuscular mycorrhizal fungi in sunflower (Helianthus annuus L.) roots
Source: Sci Rep. 2018 Jan 8;8:4. doi: 10.1038/s41598-017-18445-0 (PMC5758643; doi:10.1038/s41598-017-18445-0)
Supplement: Supplementary file 1 — Supplementary information [file 41598_2017_18445_MOESM1_ESM.pdf]

Transcriptome changes induced by arbuscular mycorrhizal fungi in sunflower (Helianthus annuus L.) roots

Alberto Vangelisti[1], Lucia Natali[1], Rodolfo Bernardi[1], Cristiana Sbrana[2], Alessandra Turrini[1], Keywan Hassani-Pak[3], David Hughes[3], Andrea Cavallini[1], Manuela Giovannetti[1], Tommaso Giordani[1]*

[1]Department of Agriculture, Food, and Environment, University of Pisa, Via del Borghetto 80, I-56124 Pisa, Italy

[2]CNR, Institute of Agricultural Biology and Biotechnology UOS Pisa, Pisa, Italy

[3]Rothamsted Research, Harpenden, Hertfordshire, AL5 2JQ

*Author for correspondence: tommaso.giordani@unipi.it +390502216671

Supplementary Fig. 1 Volcano plot of mycorrhizal versus control roots 4 days (D4) and 16 days (D16) after treatment. Red dots represent differentially expressed genes with LogFC>1 and FDR corrected pValue <0,05. LogFC= log2 Fold Change, FDR= False Discovery Rate.

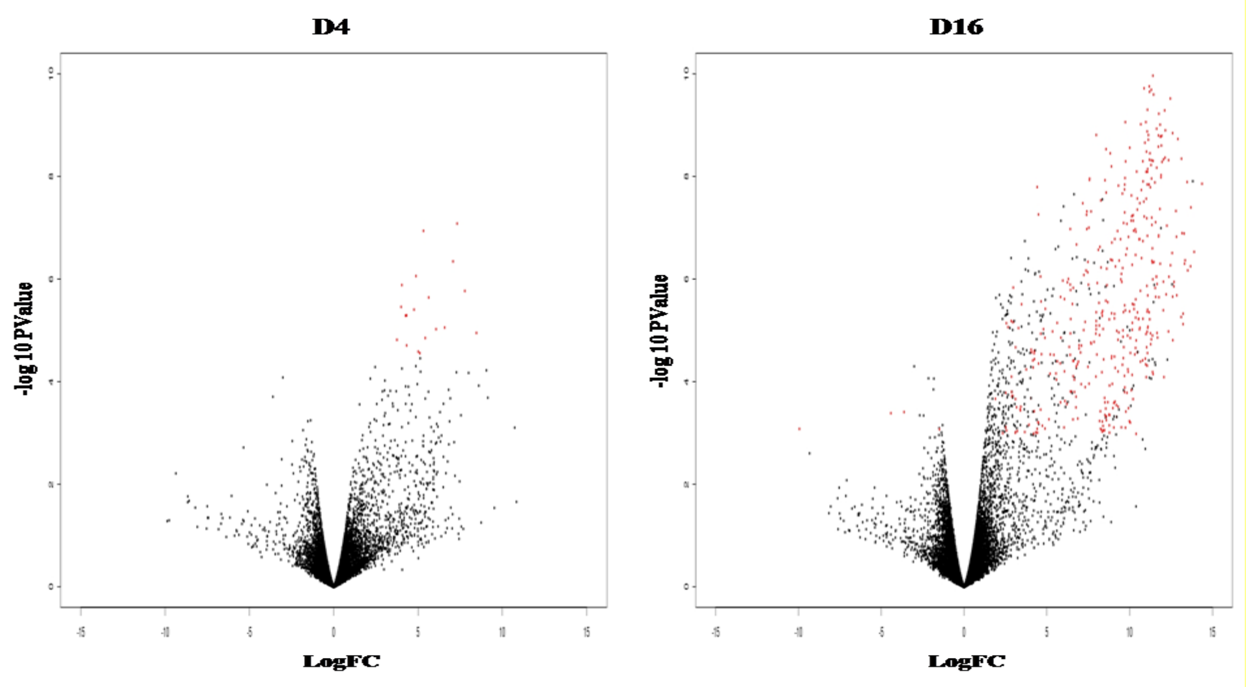

Supplementary Fig. 2 qRT-PCR analysis of five genes differentially expressed between M4 and M16 samples. The expression level of each gene was quantified by SYBR Green-based qRT-PCR using the ∆∆CT method. Data shown are fold changes calculated as transcript levels in M16 samples compared with M4 samples. Expression value of M4 samples is defined as 1. Data represent averages of three biological replicates. SD bars are indicated, * P < 0.05, ** P >0.01, *** P >0.001 .

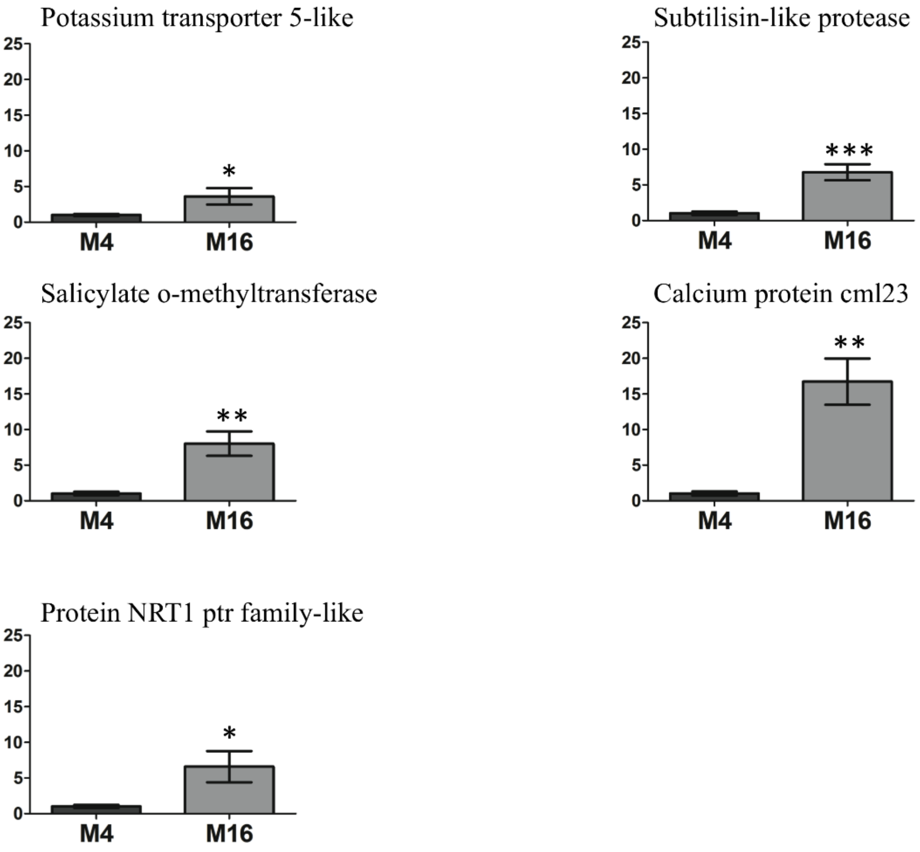

Supplementary Tab. 1 List of differentially expressed genes of H.annuus roots after 4 and 16 days from inoculation with R. irregulare. Genes with log Fold Change over 11 are blue underlined D4= Control plant versus mycorrhizal plant after 4 days, D16= Control plant versus mycorrhizal plant after 16 days, logFC= log2Fold Change, FDR= False Discovery Rate, UP= differentially over-expressed genes, DOWN= differentially under-expressed genes.

Genes identifier	logFC	FDR corrected P value	Transcript description	pairwise comparison	UP/DOWN regulation
**HanXRQChr12g0354981**	14,37	7,39E-06	Rhicadhesin receptor precursor,	D16	UP
**HanXRQChr09g0246291**	13,89	7,24E-05	pelargonidin 3-O-(6-caffeoylglucoside) 5-O-(6-O-malonylglucoside) 4 -malonyltransferase-like	D16	UP
**HanXRQChr03g0071041**	13,81	7,05E-06	senescence-specific cysteine protease SAG39-like	D16	UP
**HanXRQChr03g0072301**	13,7	1,61E-05	serine carboxypeptidase II-3-like	D16	UP
**HanXRQChr10g0308491**	13,68	0,000117502	ribulose bisphosphate carboxylase small chain clone 512-like	D16	UP
**HanXRQChr12g0375411**	13,51	0,000136114	PHR1-LIKE 1-like	D16	UP
**HanXRQChr04g0123611**	13,47	7,13E-06	germin 9-3	D16	UP
**HanXRQChr13g0405231**	13,35	9,73E-05	homogentisate phytyltransferase 1, chloroplastic isoform X2	D16	UP
**HanXRQChr16g0532281**	13,31	0,000106834	reticuline oxidase	D16	UP
**HanXRQChr03g0087431**	13,29	3,76E-05	mavicyanin-like	D16	UP
**HanXRQChr17g0542821**	13,23	0,000606516	senescence-specific cysteine protease SAG39	D16	UP

**HanXRQChr17g0542931**	13,22	0,000863116	senescence-specific cysteine protease SAG39	D16	UP
**HanXRQChr03g0087421**	13,17	3,73E-05	mavicyanin-like	D16	UP
**HanXRQChr15g0491101**	13,13	4,04E-06	subtilisin-like protease	D16	UP
**HanXRQChr03g0074461**	13,11	0,000678007	EG45-like domain containing	D16	UP
**HanXRQChr08g0218361**	12,91	2,57E-06	monocopper oxidase SKU5	D16	UP
**HanXRQChr14g0455781**	12,84	0,000321956	monocopper oxidase SKU5	D16	UP
**HanXRQChr02g0056411**	12,82	4,19E-05	AP2-like ethylene-responsive transcription factor At1g16060	D16	UP
**HanXRQChr03g0069811**	12,79	1,82E-05	pelargonidin 3-O-(6-caffeoylglucoside) 5-O-(6-O-malonylglucoside) 4 -malonyltransferase-like	D16	UP
**HanXRQChr17g0565341**	12,68	0,000204398	cytochrome P450 CYP72A219-like	D16	UP
**HanXRQChr02g0050491**	12,67	0,000233578	vinorine synthase	D16	UP
**HanXRQChr17g0542911**	12,6	0,001518398	senescence-specific cysteine protease SAG39	D16	UP
**HanXRQChr17g0542921**	12,59	0,000337209	senescence-specific cysteine protease SAG39	D16	UP
**HanXRQChr14g0458421**	12,59	2,44E-06	Subtilisin-like serine endopeptidase family	D16	UP
**HanXRQChr13g0405221**	12,57	7,57E-05	feruloyl ortho-hydroxylase 1-like	D16	UP
**HanXRQChr17g0542901**	12,56	0,000221146	vignain-like	D16	UP

**HanXRQChr13g0409781**	12,46	0,00011297	beta-amyrin 28-oxidase-like	D16	UP
**HanXRQChr11g0350641**	12,45	1,44E-06	UDP-glycosyltransferase 73C3-like	D16	UP
**HanXRQChr05g0159491**	12,37	0,000169863	GDSL esterase lipase At5g55050-like	D16	UP
**HanXRQChr08g0230271**	12,35	4,04E-06	PREDICTED: uncharacterized protein LOC104086504	D16	UP
**HanXRQChr16g0521981**	12,34	0,001116416	ripening-related 1	D16	UP
**HanXRQChr08g0209751**	12,28	0,003115299	senescence-specific cysteine protease SAG39	D16	UP
**HanXRQChr17g0546161**	12,25	2,89E-05	MO25 At5g47540	D16	UP
**HanXRQChr17g0556671**	12,2	0,000312981	cytochrome P450 CYP72A219-like	D16	UP
**HanXRQChr09g0244301**	12,15	2,44E-06	unnamed protein product	D16	UP
**HanXRQChr07g0194061**	12,14	4,04E-06	germin 9-3	D16	UP
**HanXRQChr06g0168801**	12,11	1,91E-06	germin 9-3	D16	UP
**HanXRQChr10g0309481**	12,07	0,005871305	beta-amyrin 28-oxidase-like	D16	UP
**HanXRQChr05g0150391**	12,04	4,04E-06	senescence-specific cysteine protease SAG39-like	D16	UP
**HanXRQChr08g0237031**	12,03	0,000204079	ATP-dependent RNA helicase dbp6	D16	UP
**HanXRQChr01g0023211**	12,03	0,00054856	cell number regulator 10-like	D16	UP
**HanXRQChr03g0072291**	11,95	4,04E-06	serine carboxypeptidase II-3-like	D16	UP
**HanXRQChr08g0224831**	11,93	2,44E-06	probable ascorbate-specific	D16	UP

			transmembrane electron transporter 1		
**HanXRQChr03g0067111**	11,91	3,08E-06	beta-amyrin 28-oxidase-like	D16	UP
**HanXRQChr03g0087951**	11,9	6,17E-06	Vacuolar-processing enzyme precursor	D16	UP
**HanXRQChr05g0144081**	11,89	6,67E-06	inorganic phosphate transporter 1-11-like	D16	UP
**HanXRQChr13g0403541**	11,89	3,17E-05	lysosomal Pro-X carboxypeptidase-like	D16	UP
**HanXRQChr13g0417231**	11,86	2,44E-06	vacuolar-processing enzyme beta-isozyme	D16	UP
**HanXRQChr14g0456101**	11,84	0,000106834	beta-amyrin 28-oxidase-like	D16	UP
**HanXRQChr07g0192561**	11,84	2,33E-06	GDSL esterase lipase At5g55050-like	D16	UP
**HanXRQChr03g0065351**	11,83	1,71E-05	transmembrane 9 superfamily member 4-like	D16	UP
**HanXRQChr08g0224691**	11,76	0,000339336	clathrin assembly At2g25430	D16	UP
**HanXRQChr03g0087411**	11,76	2,00E-06	mavicyanin-like	D16	UP
**HanXRQChr05g0161981**	11,75	4,06E-05	citrate-binding -like	D16	UP
**HanXRQChr13g0417181**	11,75	4,18E-05	Early nodulin 55-2 precursor,	D16	UP
**HanXRQChr14g0458411**	11,74	2,44E-06	Cucumisin precursor,	D16	UP
**HanXRQChr13g0417121**	11,73	3,13E-06	vacuolar-processing enzyme beta-isozyme-like	D16	UP
**HanXRQChr12g0375911**	11,68	0,001378366	BAHD acyltransferase At5g47980-like	D16	UP

**HanXRQChr13g0417191**	11,65	0,000218835	Uclacyanin 1,	D16	UP
**HanXRQChr03g0065321**	11,65	0,001164701	endomembrane 70	D16	UP
**HanXRQChr16g0522021**	11,63	4,90E-06	ripening-related 1	D16	UP
**HanXRQChr07g0196931**	11,63	2,44E-06	AMP-dependent synthetase and ligase family	D16	UP
**HanXRQChr09g0262051**	11,61	0,000169098	DUF21 domain-containing At2g14520-like	D16	UP
**HanXRQChr04g0123561**	11,61	0,000169863	replication factor C subunit 3-like	D16	UP
**HanXRQChr17g0558311**	11,57	0,000337231	core-2 I-branching enzyme	D16	UP
**HanXRQChr16g0517561**	11,56	2,57E-05	conserved hypothetical protein	D16	UP
**HanXRQChr04g0112811**	11,55	0,003684698	chitinase family	D16	UP
**HanXRQChr08g0211571**	11,54	0,000246208	probable mitochondrial-processing peptidase subunit beta	D16	UP
**HanXRQChr04g0112801**	11,53	0,003395021	chitinase family	D16	UP
**HanXRQChr16g0503641**	11,52	7,17E-06	probable inactive receptor kinase At2g26730	D16	UP
**HanXRQChr13g0405261**	11,48	0,000223187	glycinol 4-dimethylallyltransferase-like	D16	UP
**HanXRQChr05g0140501**	11,46	0,000102013	probable 2-oxoglutarate-dependent dioxygenase AOP1	D16	UP
**HanXRQChr03g0074171**	11,44	1,41E-06	unnamed protein product	D16	UP
**HanXRQChr02g0040391**	11,44	0,000405984	endoglucanase 25-like	D16	UP

**HanXRQChr04g0096201**	11,41	4,04E-06	NRT1 PTR FAMILY	D16	UP
**HanXRQChr03g0063471**	11,41	0,005219071	UPF0481 At3g02645	D16	UP
**HanXRQChr15g0472261**	11,4	1,41E-06	NRT1 PTR FAMILY -like	D16	UP
**HanXRQChr08g0235831**	11,39	0,000186868	Beta-3 adrenergic receptor,	D16	UP
**HanXRQChr04g0127761**	11,38	0,004836329	purple acid phosphatase	D16	UP
**HanXRQChr04g0126361**	11,38	0,003059273	costunolide synthase	D16	UP
**HanXRQChr03g0073511**	11,38	0,001271389	subtilisin-like protease	D16	UP
**HanXRQChr10g0283371**	11,37	0,000103185	zeatin O-glucosyltransferase-like	D16	UP
**HanXRQChr11g0334031**	11,37	6,42E-05	probable inactive receptor kinase At4g23740	D16	UP
**HanXRQChr09g0266451**	11,34	9,86E-05	isoflavone 2 -hydroxylase-like	D16	UP
**HanXRQChr09g0249591**	11,34	2,82E-05	probable inactive receptor kinase At2g26730	D16	UP
**HanXRQChr03g0087401**	11,33	2,23E-05	plastocyanin-like domain	D16	UP
**HanXRQChr10g0302921**	11,32	6,71E-06	probable 2-oxoglutarate-dependent dioxygenase AOP1	D16	UP
**HanXRQChr08g0232451**	11,31	4,04E-06	transmembrane ,	D16	UP
**HanXRQChr13g0397191**	11,31	0,001466428	germin 9-3	D16	UP
**HanXRQChr03g0074111**	11,3	1,41E-06	unnamed protein product	D16	UP
**HanXRQChr05g0134041**	11,3	0,006166169	ABC transporter B family member 19	D16	UP
**HanXRQChr11g0320051**	11,29	4,53E-06	abscisic acid 8 -hydroxylase 3-like	D16	UP

**HanXRQChr05g0133861**	11,27	0,001762651	chitinase 2-like	D16	UP
**HanXRQChr03g0060091**	11,25	0,001175872	ankyrin repeat-containing At3g12360-like isoform X1	D16	UP
**HanXRQChr09g0275631**	11,25	7,97E-06	clavaminate synthase At3g21360	D16	UP
**HanXRQChr08g0217301**	11,22	3,58E-06	germin 9-2	D16	UP
**HanXRQChr08g0209761**	11,21	0,000727822	senescence-specific cysteine protease SAG39	D16	UP
**HanXRQChr13g0419211**	11,2	1,41E-06	subtilisin-like protease	D16	UP
**HanXRQChr06g0168781**	11,2	4,04E-06	germin 9-2	D16	UP
**HanXRQChr17g0542891**	11,2	0,006006827	senescence-specific cysteine protease SAG39	D16	UP
**HanXRQChr04g0127841**	11,18	1,41E-06	ammonium transporter 3 member 1-like	D16	UP
**HanXRQChr16g0521951**	11,18	0,000580779	ripening-related 1	D16	UP
**HanXRQChr09g0256471**	11,17	7,39E-06	DELLA RGL1-like	D16	UP
**HanXRQChr17g0542851**	11,15	0,003200079	senescence-specific cysteine protease SAG39	D16	UP
**HanXRQChr06g0168791**	11,13	2,44E-06	germin 9-2	D16	UP
**HanXRQChr16g0532301**	11,13	2,57E-06	probable glutathione S-transferase	D16	UP
**HanXRQChr03g0069511**	11,1	0,000956788	PREDICTED: uncharacterized protein LOC104221946	D16	UP
**HanXRQChr13g0405241**	11,09	0,000321956	homogentisate phytyltransferase 1, chloroplastic isoform X1	D16	UP

**HanXRQChr17g0535951**	11,09	0,000991213	Palmate-like pentafoliata 1 transcription factor	D16	UP
**HanXRQChr04g0116251**	11,08	5,09E-06	aquaporin NIP1-1-like	D16	UP
**HanXRQChr04g0112821**	11,08	0,000539836	hevamine-A-like	D16	UP
**HanXRQChr17g0557171**	11,08	5,42E-06	ABC transporter family	D16	UP
**HanXRQChr10g0285621**	11,08	0,002450691	ATP binding cassette subfamily B4 isoform 2	D16	UP
**HanXRQChr08g0216661**	11,08	1,91E-06	inorganic phosphate transporter 1-11-like	D16	UP
**HanXRQChr04g0115941**	11,07	4,09E-05	PREDICTED: uncharacterized protein LOC102602677	D16	UP
**HanXRQChr13g0419221**	11,06	7,39E-06	subtilisin-like protease	D16	UP
**HanXRQChr07g0189611**	11,06	0,000572687	SRG1-like	D16	UP
**HanXRQChr10g0280991**	11,06	2,44E-06	inorganic phosphate transporter 1-4-like isoform X2	D16	UP
**HanXRQChr08g0211591**	11,05	0,00380907	probable mitochondrial-processing peptidase subunit beta	D16	UP
**HanXRQChr02g0050761**	11,05	0,001124479	ammonium transporter 3 member 1-like	D16	UP
**HanXRQChr03g0074321**	11,04	0,00023157	EG45-like domain containing	D16	UP
**HanXRQChr07g0192551**	11,03	2,27E-05	---NA---	D16	UP
**HanXRQChr13g0403551**	11,02	0,001982439	lysosomal Pro-X carboxypeptidase-like	D16	UP

**HanXRQChr01g0024211**	11,01	3,53E-06	subtilisin-like protease	D16	UP
**HanXRQChr03g0074451**	11	0,005871305	EG45-like domain containing	D16	UP
**HanXRQChr10g0288161**	11	1,19E-05	cysteine ase RD21a-like	D16	UP
**HanXRQChr09g0246281**	11	5,42E-06	pelargonidin 3-O-(6-caffeoylglucoside) 5-O-(6-O-malonylglucoside) 4 -malonyltransferase-like	D16	UP
**HanXRQChr03g0074281**	10,99	0,001195351	EG45-like domain containing	D16	UP
**HanXRQChr05g0159761**	10,98	2,33E-06	chitinase 2-like	D16	UP
**HanXRQChr10g0307331**	10,97	0,00362063	DELLA RGL1-like	D16	UP
**HanXRQChr06g0176051**	10,95	0,003016517	chitinase 2-like	D16	UP
**HanXRQChr04g0115981**	10,92	2,82E-06	senescence-specific cysteine protease SAG39-like	D16	UP
**HanXRQChr07g0187871**	10,88	2,14E-05	(+)-neomenthol dehydrogenase-like	D16	UP
**HanXRQChr03g0087471**	10,87	1,41E-06	Subtilisin-like protease SDD1	D16	UP
**HanXRQChr03g0065341**	10,85	1,31E-05	transmembrane 9 superfamily member 4-like	D16	UP
**HanXRQChr03g0073501**	10,81	7,05E-06	subtilisin-like protease	D16	UP
**HanXRQChr02g0035401**	10,8	0,000723252	cytochrome P450 71A6-like	D16	UP
**HanXRQChr04g0097831**	10,8	4,81E-05	probable serine threonine-kinase DDB_G0267514	D16	UP
**HanXRQChr15g0488781**	10,8	4,04E-06	UDP-glycosyltransferase 83A1-like	D16	UP

**HanXRQChr13g0398851**	10,78	0,003200079	Uclacyanin 1,	D16	UP
**HanXRQChr05g0147741**	10,72	9,18E-05	germin 9-2	D16	UP
**HanXRQChr15g0488511**	10,71	8,74E-06	glycerol-3-phosphate 2-O-acyltransferase 6-like	D16	UP
**HanXRQChr13g0407131**	10,67	2,33E-06	neutral ceramidase-like	D16	UP
**HanXRQChr15g0497021**	10,65	0,003309288	mitochondrial import inner membrane translocase subunit TIM22	D16	UP
**HanXRQChr13g0417651**	10,65	0,000273394	butyrate-- ligase AAE11, peroxisomal-like	D16	UP
**HanXRQChr07g0194471**	10,62	0,000993128	calcium-dependent kinase 1-like	D16	UP
**HanXRQChr01g0024241**	10,61	1,37E-05	Subtilisin-like protease	D16	UP
**HanXRQChr06g0183411**	10,6	4,51E-05	probable glutathione S-transferase	D16	UP
**HanXRQChr17g0558071**	10,59	0,001041473	rop guanine nucleotide exchange factor 14	D16	UP
**HanXRQChr01g0024221**	10,57	4,51E-05	Subtilisin-like protease	D16	UP
**HanXRQChr02g0048531**	10,55	0,000723252	DELLA RGL1-like	D16	UP
**HanXRQChr03g0074271**	10,53	0,000724219	EG45-like domain containing	D16	UP
**HanXRQChr13g0414681**	10,49	0,002376495	endoglucanase 24-like	D16	UP
**HanXRQChr16g0517841**	10,47	0,000582012	kelch 8	D16	UP
**HanXRQChr05g0136221**	10,46	1,19E-05	senescence-specific cysteine protease SAG39-like	D16	UP
**HanXRQChr06g0171251**	10,46	1,74E-05	probable S-adenosylmethionine-	D16	UP

			dependent methyltransferase At5g37990		
**HanXRQChr08g0228941**	10,45	0,002409267	NADPH oxidase	D16	UP
**HanXRQChr04g0096381**	10,42	5,42E-06	neurogenic locus delta-like isoform X2	D16	UP
**HanXRQChr12g0362001**	10,39	0,001527973	ethylene-responsive transcription factor RAP2-2-like	D16	UP
**HanXRQChr13g0403461**	10,39	0,049236978	beta-galactosidase 8	D16	UP
**HanXRQChr03g0065331**	10,37	3,52E-05	transmembrane 9 superfamily member 4-like	D16	UP
**HanXRQChr04g0113901**	10,37	0,000368697	ammonium transporter 3 member 1-like	D16	UP
**HanXRQChr02g0048521**	10,36	0,000696058	DELLA RGL1-like	D16	UP
**HanXRQChr08g0212191**	10,36	8,12E-05	serine carboxypeptidase-like 51	D16	UP
**HanXRQChr03g0063121**	10,36	0,002033437	receptor kinase HERK 1	D16	UP
**HanXRQChr04g0112841**	10,34	3,25E-05	chitinase family	D16	UP
**HanXRQChr13g0426381**	10,31	0,002568282	ripening-related 1	D16	UP
**HanXRQChr13g0414221**	10,26	0,000438753	beta-amyrin synthase	D16	UP
**HanXRQChr14g0445851**	10,26	0,00052548	LURP-one-related 5-like	D16	UP
**HanXRQChr17g0552681**	10,23	3,43E-05	triose phosphate phosphate translocator, chloroplastic-like	D16	UP
**HanXRQChr17g0547871**	10,23	0,008064068	Serine-threonine kinase,	D16	UP
**HanXRQChr14g0455791**	10,22	2,44E-05	monocopper oxidase SKU5	D16	UP

**HanXRQChr02g0038171**	10,21	2,06E-05	subtilisin-like protease	D16	UP
**HanXRQChr05g0156451**	10,21	0,013527439	NRT1 PTR FAMILY -like	D16	UP
**HanXRQChr10g0287371**	10,17	0,000432937	NRT1 PTR FAMILY -like	D16	UP
**HanXRQChr14g0444211**	10,16	0,000826009	L-type lectin-domain containing receptor kinase -like	D16	UP
**HanXRQChr17g0569201**	10,16	0,001058792	S-adenosyl-L-methionine-dependent methyltransferases superfamily	D16	UP
**HanXRQChr05g0150371**	10,15	0,000111331	senescence-specific cysteine protease SAG39-like	D16	UP
**HanXRQChr07g0197681**	10,15	0,009145006	inactive kinase SELMODRAFT_444075-like	D16	UP
**HanXRQChr13g0400501**	10,15	0,005387658	sporulation-specific 15-like	D16	UP
**HanXRQChr04g0102951**	10,13	2,14E-05	reticuline oxidase-like	D16	UP
**HanXRQChr15g0490031**	10,11	1,06E-05	nodulin-26-like	D16	UP
**HanXRQChr13g0426391**	10,1	0,009699572	ripening-related 1	D16	UP
**HanXRQChr04g0112881**	10,09	2,44E-05	chitinase family	D16	UP
**HanXRQChr12g0365471**	10,06	0,017693757	G-type lectin S-receptor-like serine threonine- kinase	D16	UP
**HanXRQChr02g0052351**	10,05	0,002448047	cyclic nucleotide-gated ion channel 15	D16	UP
**HanXRQChr17g0544331**	10,04	0,00138975	probable transcription factor 21	D16	UP
**HanXRQChr16g0509291**	10,03	0,0326408	ras-related RIC2	D16	UP

**HanXRQChr03g0074301**	10	0,001071786	EG45-like domain containing	D16	UP
**HanXRQChr17g0558201**	10	3,17E-06	oligopeptide transporter 1-like	D16	UP
**HanXRQChr05g0139591**	9,99	0,000569828	GDSL esterase lipase At5g55050-like	D16	UP
**HanXRQChr11g0348851**	9,98	0,031029827	major allergen Pru ar 1-like	D16	UP
**HanXRQChr17g0534261**	9,93	4,19E-05	embryogenesis-associated EMB8-like	D16	UP
**HanXRQChr10g0309181**	9,93	0,019178778	homogentisate phytyltransferase 1, chloroplastic isoform X1	D16	UP
**HanXRQChr03g0071791**	9,9	0,004784942	Proline-rich receptor kinase PERK10	D16	UP
**HanXRQChr11g0348651**	9,89	0,004357425	major allergen Pru ar 1-like	D16	UP
**HanXRQChr17g0557611**	9,87	0,001273056	PI-PLC X-box domain-containing DDB_G0293730-like	D16	UP
**HanXRQChr17g0542841**	9,86	0,000961724	senescence-specific cysteine protease SAG39	D16	UP
**HanXRQChr03g0081281**	9,84	6,45E-05	probable LRR receptor-like serine threonine- kinase At4g31250	D16	UP
**HanXRQChr04g0119111**	9,83	0,010681455	inactive kinase SELMODRAFT_444075-like	D16	UP
**HanXRQChr16g0513511**	9,83	2,71E-05	peroxidase 5-like	D16	UP
**HanXRQChr15g0488981**	9,77	8,11E-05	Subtilisin-like serine endopeptidase family	D16	UP

**HanXRQChr10g0278141**	9,77	0,021690786	wall-associated receptor kinase 2-like	D16	UP
**HanXRQChr15g0492721**	9,75	4,04E-06	vinorine synthase-like	D16	UP
**HanXRQChr11g0348731**	9,74	0,039432459	major allergen Pru ar 1-like	D16	UP
**HanXRQChr13g0395591**	9,74	0,00054856	DELLA RGL1-like	D16	UP
**HanXRQChr05g0158211**	9,74	2,33E-06	hydroxycinnamoyl-shikimate quinate hydroxycinnamoyltransferase	D16	UP
**HanXRQChr12g0375201**	9,72	6,71E-06	ABC transporter G family member 17-like	D16	UP
**HanXRQChr02g0042121**	9,71	0,008667669	jacalin-related lectin 3-like	D16	UP
**HanXRQChr17g0546611**	9,7	7,39E-06	UDP-glycosyltransferase 83A1	D16	UP
**HanXRQChr11g0337041**	9,7	1,88E-05	premnaspirodiene oxygenase-like	D16	UP
**HanXRQChr13g0400111**	9,7	0,000449535	cyclic nucleotide-gated ion channel 15	D16	UP
**HanXRQChr16g0509551**	9,69	8,66E-06	plasma membrane ATPase 1	D16	UP
**HanXRQChr16g0513331**	9,69	0,005619282	bidirectional sugar transporter SWEET16-like	D16	UP
**HanXRQChr10g0277951**	9,69	0,010377628	wall-associated receptor kinase 2-like	D16	UP
**HanXRQChr15g0482321**	9,64	0,00197868	abscisic acid 8 -hydroxylase 3-like	D16	UP
**HanXRQChr14g0447371**	9,64	0,009699572	wall-associated receptor kinase-like 1	D16	UP

**HanXRQChr09g0243561**	9,63	2,64E-05	BAHD acyltransferase At5g47980-like	D16	UP
**HanXRQChr17g0565301**	9,62	0,000580601	RING-H2 finger ATL51-like	D16	UP
**HanXRQChr12g0357401**	9,62	0,002962986	serine carboxypeptidase-like 45	D16	UP
**HanXRQChr17g0560681**	9,6	0,001334117	gibberellin 20-oxidase	D16	UP
**HanXRQChr13g0426441**	9,59	0,001071786	ripening-related 1	D16	UP
**HanXRQChr13g0400381**	9,58	0,000124744	UDP-glycosyltransferase 73C3	D16	UP
**HanXRQChr06g0170841**	9,56	0,003674179	cytochrome P450 CYP72A219-like	D16	UP
**HanXRQChr08g0209131**	9,56	9,99E-06	glycerol-3-phosphate 2-O-acyltransferase 6-like	D16	UP
**HanXRQChr02g0037171**	9,52	0,000204398	probable inactive receptor kinase At2g26730	D16	UP
**HanXRQChr13g0407121**	9,5	0,000153496	neutral ceramidase-like	D16	UP
**HanXRQChr06g0172971**	9,5	0,039023344	5 -AMP-activated kinase subunit gamma-3,	D16	UP
**HanXRQChr12g0355491**	9,47	0,001591436	ras-related RHN1-like	D16	UP
**HanXRQChr10g0319901**	9,47	0,000186868	L-type lectin-domain containing receptor kinase -like	D16	UP
**HanXRQChr17g0537681**	9,45	4,51E-05	5 -AMP-activated kinase subunit gamma-3,	D16	UP
**HanXRQChr13g0410281**	9,36	9,77E-05	deacetylvindoline O-acetyltransferase-like	D16	UP

**HanXRQChr04g0105851**	9,35	0,01998287	nodulation-signaling pathway 2 -like	D16	UP
**HanXRQChr03g0084241**	9,34	0,001148406	PREDICTED: uncharacterized protein LOC105140517	D16	UP
**HanXRQChr03g0072271**	9,32	7,63E-06	serine carboxypeptidase II-3-like	D16	UP
**HanXRQChr03g0074181**	9,3	0,01820974	unnamed protein product	D16	UP
**HanXRQChr01g0014321**	9,3	0,004734927	DELLA RGL1-like	D16	UP
**HanXRQChr11g0348801**	9,3	0,001492534	major allergen Pru ar 1-like	D16	UP
**HanXRQChr05g0139341**	9,27	3,45E-05	AP2-like ethylene-responsive transcription factor At1g16060	D16	UP
**HanXRQChr08g0216331**	9,22	0,026211496	probable inorganic phosphate transporter 1-9	D16	UP
**HanXRQChr16g0522011**	9,22	0,001093937	ripening-related 1	D16	UP
**HanXRQChr07g0201571**	9,21	0,026150592	Cucumisin precursor,	D16	UP
**HanXRQChr03g0092331**	9,19	0,01722459	MATH domain-containing At5g43560 isoform X1	D16	UP
**HanXRQChr13g0410291**	9,14	0,00011789	Salutaridinol 7-O-acetyltransferase	D16	UP
**HanXRQChr10g0303401**	9,12	0,004114784	Serine-threonine kinase, plant-type,	D16	UP
**HanXRQChr03g0087461**	9,11	0,015076378	mavicyanin-like	D16	UP
**HanXRQChr04g0120381**	9,1	4,51E-05	Cytochrome B5, n2,ATCB5-E,CB5-E isoform 1	D16	UP

**HanXRQChr10g0304741**	9,09	0,000607502	Sodium calcium exchanger family calcium-binding EF hand family isoform 1	D16	UP
**HanXRQChr05g0131191**	9,09	0,002786483	hypothetical protein VITISV_011098	D16	UP
**HanXRQChr10g0277941**	9,08	0,000715437	wall-associated receptor kinase 2-like	D16	UP
**HanXRQChr03g0070861**	9,05	0,025834198	probable leucine-rich repeat receptor kinase At5g49770	D16	UP
**HanXRQChr15g0474681**	9,05	0,000231971	lysine histidine transporter 1-like	D16	UP
**HanXRQChr10g0315791**	9,04	0,039323986	beta-1,4-mannosyl-glyco 4-beta-N-acetylglucosaminyltransferase	D16	UP
**HanXRQChr01g0019511**	9,04	0,015882443	lactoylglutathione lyase	D16	UP
**HanXRQChr08g0216351**	9	0,028670945	probable inorganic phosphate transporter 1-9	D16	UP
**HanXRQChr09g0242701**	8,99	0,019141823	probable serine threonine-kinase Cx32, chloroplastic	D16	UP
**HanXRQChr03g0071781**	8,95	0,007549425	receptor-like cytosolic serine threonine- kinase RBK2	D16	UP
**HanXRQChr03g0080671**	8,95	9,73E-05	tetrahydrocannabinolic acid synthase-like	D16	UP
**HanXRQChr04g0115991**	8,92	5,10E-05	senescence-specific cysteine protease SAG39-like	D16	UP
**HanXRQChr15g0488611**	8,92	0,002757313	hypothetical protein VITISV_011099	D16	UP

**HanXRQChr06g0165261**	8,91	0,000601111	geraniol 8-hydroxylase-like	D16	UP
**HanXRQChr03g0067771**	8,91	0,00296388	EG45-like domain containing	D16	UP
**HanXRQChr13g0396481**	8,9	4,72E-06	AP2-like ethylene-responsive transcription factor At1g16060	D16	UP
**HanXRQChr11g0325471**	8,86	9,62E-05	ATPase family associated with various cellular activities,	D16	UP
**HanXRQChr13g0398831**	8,83	3,58E-06	stellacyanin-like	D16	UP
**HanXRQChr15g0470741**	8,79	0,024624593	heat shock 70 kDa 16	D16	UP
**HanXRQChr10g0291521**	8,79	0,008050238	nodulation-signaling pathway 2 -like	D16	UP
**HanXRQChr10g0318721**	8,78	0,046987421	E3 ubiquitin- ligase LIN-1	D16	UP
**HanXRQChr10g0309121**	8,77	0,000727872	short-chain dehydrogenase TIC 32, chloroplastic-like	D16	UP
**HanXRQChr15g0463231**	8,77	0,036370554	heat shock cognate 70 kDa 2-like	D16	UP
**HanXRQChr08g0227341**	8,75	0,024371362	laccase-1-like	D16	UP
**HanXRQChr12g0362771**	8,68	0,015436044	Malectin receptor kinase family	D16	UP
**HanXRQChr11g0321251**	8,68	0,000405984	pleiotropic drug resistance 2-like	D16	UP
**HanXRQChr10g0302151**	8,68	0,000102013	probable 2-oxoglutarate-dependent dioxygenase AOP1	D16	UP
**HanXRQChr16g0532951**	8,63	0,000225241	major allergen Pru ar 1-like	D16	UP

**HanXRQChr11g0339021**	8,62	0,000127623	Late embryogenesis abundant hydroxyproline-rich glyco family,	D16	UP
**HanXRQChr08g0235961**	8,59	0,029681574	acyltransferase At1g54570, chloroplastic isoform X1	D16	UP
**HanXRQChr16g0526361**	8,59	0,025829214	RING-H2 finger ATL51-like	D16	UP
**HanXRQChr03g0072261**	8,58	9,69E-06	serine carboxypeptidase II-3-like	D16	UP
**HanXRQChr08g0213691**	8,57	3,26E-06	cytochrome P450 CYP72A219-like	D16	UP
**HanXRQChr12g0365411**	8,56	0,000389043	cytochrome P450 reductase	D16	UP
**HanXRQChr11g0325281**	8,55	0,019435473	transmembrane ,	D16	UP
**HanXRQChr01g0019871**	8,55	0,026768338	probable glutathione S-transferase	D16	UP
**HanXRQChr09g0265501**	8,54	0,000237175	AP2-like ethylene-responsive transcription factor At1g16060	D16	UP
**HanXRQChr04g0112311**	8,51	6,93E-06	NRT1 PTR FAMILY -like	D16	UP
**HanXRQChr11g0325291**	8,51	0,015492839	Integrin-linked kinase family	D16	UP
**HanXRQChr12g0370001**	8,5	0,024624593	RING-H2 finger ATL52-like	D16	UP
**HanXRQChr12g0365931**	8,49	0,01301108	ribulose bisphosphate carboxylase small chain clone 512-like	D16	UP
**HanXRQChr05g0137371**	8,47	0,043331138	subtilisin-like protease	D16	UP
**HanXRQChr08g0222221**	8,44	0,043584542	Polyketide cyclase dehydrase and lipid	D16	UP

			transport superfamily		
**HanXRQChr11g0353641**	8,43	0,003085947	probable polygalacturonase At3g15720	D16	UP
**HanXRQChr07g0187861**	8,4	2,44E-05	(+)-neomenthol dehydrogenase-like	D16	UP
**HanXRQChr10g0319891**	8,4	0,001035583	squalene monooxygenase-like	D16	UP
**HanXRQChr04g0106871**	8,39	0,0370766	RING-H2 finger ATL66-like	D16	UP
**HanXRQChr06g0175171**	8,37	0,000560978	transaldolase family	D16	UP
**HanXRQChr13g0400491**	8,36	6,97E-05	F-box and Leucine Rich Repeat domains containing , isoform 2	D16	UP
**HanXRQChr01g0016411**	8,35	1,21E-05	probable inactive purple acid phosphatase 27	D16	UP
**HanXRQChr10g0283921**	8,35	0,031335009	zinc finger NUTCRACKER-like	D16	UP
**HanXRQChr14g0442011**	8,34	0,000646476	probable prolyl 4-hydroxylase 10	D16	UP
**HanXRQChr14g0430011**	8,34	0,04552961	heparan-alpha-glucosaminide N-acetyltransferase-like	D16	UP
**HanXRQChr07g0189621**	8,32	0,000597587	SRG1-like	D16	UP
**HanXRQChr06g0165131**	8,32	0,004853028	molybdenum cofactor sulfurase-like	D16	UP
**HanXRQChr10g0307171**	8,32	0,043340669	BAHD acyltransferase At5g47980-like	D16	UP
**HanXRQChr13g0395561**	8,27	0,000122459	DELLA RGL1-like	D16	UP

**HanXRQChr04g0112831**	8,26	1,25E-05	hevamine-A-like	D16	UP
**HanXRQChr07g0194001**	8,24	0,041036941	ankyrin repeat-containing At3g12360-like isoform X3	D16	UP
**HanXRQChr05g0152781**	8,23	0,021411825	Histone	D16	UP
**HanXRQChr10g0280731**	8,2	6,06E-05	NRT1 PTR FAMILY -like	D16	UP
**HanXRQChr14g0451451**	8,19	0,000705853	probable 2-oxoglutarate-dependent dioxygenase AOP1	D16	UP
**HanXRQChr15g0483191**	8,18	0,030799304	citrate-binding -like	D16	UP
**HanXRQChr16g0517551**	8,18	0,001250767	hypothetical protein EUGRSUZ_I00207	D16	UP
**HanXRQChr11g0333441**	8,17	0,000914737	cytochrome P450 71D11-like	D16	UP
**HanXRQChr11g0337051**	8,15	0,000417241	premnaspirodiene oxygenase-like	D16	UP
**HanXRQChr05g0139291**	8,14	0,000298999	CDPK-related kinase 4-like isoform X1	D16	UP
**HanXRQChr03g0074151**	8,11	6,69E-05	unnamed protein product	D16	UP
**HanXRQChr16g0514971**	8,04	0,00021078	caffeoyl-O-methyltransferase	D16	UP
**HanXRQChr11g0320261**	8,02	0,002568282	amino acid permease 3-like	D16	UP
**HanXRQChr06g0174451**	7,98	2,44E-06	lysM domain receptor-like kinase 3	D16	UP
**HanXRQChr11g0332961**	7,98	0,001041473	multidrug resistance	D16	UP
**HanXRQChr13g0411421**	7,98	0,000580779	probably inactive leucine-rich repeat receptor kinase At1g50610	D16	UP

**HanXRQChr11g0333451**	7,98	0,000770199	cytochrome P450 family 71	D16	UP
**HanXRQChr05g0154681**	7,98	0,000914737	DELLA RGL1-like	D16	UP
**HanXRQChr13g0426401**	7,96	0,005764656	ripening-related 1	D16	UP
**HanXRQChr07g0195301**	7,96	0,001181278	cyclic nucleotide-gated ion channel 15	D16	UP
**HanXRQChr11g0321931**	7,85	0,000103765	metal tolerance 9-like	D16	UP
**HanXRQChr08g0217851**	7,85	0,001608808	pleiotropic drug resistance 2-like	D16	UP
**HanXRQChr16g0522791**	7,68	0,001722217	riboflavin biosynthesis PYRD, chloroplastic-like	D16	UP
**HanXRQChr13g0423171**	7,67	1,82E-05	plasma membrane ATPase 1	D16	UP
**HanXRQChr03g0074261**	7,66	0,001661257	EG45-like domain containing	D16	UP
**HanXRQChr11g0342651**	7,64	0,000312981	feruloyl ortho-hydroxylase 1-like	D16	UP
**HanXRQChr06g0181771**	7,58	6,71E-06	NRT1 PTR FAMILY -like	D16	UP
**HanXRQChr09g0257681**	7,57	6,93E-06	germin 2-1	D16	UP
**HanXRQChr16g0501981**	7,57	0,003395021	agmatine deiminase	D16	UP
**HanXRQChr08g0231701**	7,56	0,000253446	cytochrome b561 and DOMON domain-containing At3g25290	D16	UP
**HanXRQChr07g0201601**	7,55	0,00749274	ribulose bisphosphate carboxylase oxygenase activase, chloroplastic isoform X2	D16	UP
**HanXRQChr09g0277141**	7,54	3,14E-05	ABC transporter B family member 19-like	D16	UP

**HanXRQChr03g0090491**	7,51	0,000222551	tubulin alpha-3 chain	D16	UP
**HanXRQChr03g0074801**	7,48	0,003970042	subtilisin-like protease	D16	UP
**HanXRQChr01g0019851**	7,48	0,000102013	probable glutathione S-transferase	D16	UP
**HanXRQChr17g0546631**	7,46	0,000144895	UDP-glycosyltransferase 83A1-like	D16	UP
**HanXRQChr03g0084791**	7,42	1,82E-05	BAHD acyltransferase At5g47980-like	D16	UP
**HanXRQChr16g0497891**	7,41	3,24E-05	DELLA RGL1-like	D16	UP
**HanXRQChr15g0474021**	7,39	2,05E-05	ABC transporter G family member 17-like	D16	UP
**HanXRQChr09g0272901**	7,37	0,000136114	DELLA RGL1-like	D16	UP
**HanXRQChr05g0140521**	7,35	0,024336362	kelch 8	D16	UP
**HanXRQChr09g0246251**	7,31	0,008563836	serine carboxypeptidase	D16	UP
**HanXRQChr09g0272331**	7,31	0,000798286	probable inactive receptor kinase At2g26730	D16	UP
**HanXRQChr11g0339811**	7,29	9,63E-05	bifunctional epoxide hydrolase 2	D16	UP
**HanXRQChr12g0386221**	7,23	0,00015952	zinc finger NUTCRACKER-like	D16	UP
**HanXRQChr17g0542881**	7,22	0,001066252	senescence-specific cysteine protease SAG39	D16	UP
**HanXRQChr10g0295581**	7,17	1,37E-05	germin 2-1	D16	UP
**HanXRQChr05g0157141**	7,09	0,001856938	WEAK CHLOROPLAST MOVEMENT UNDER BLUE LIGHT 1-like	D16	UP
**HanXRQChr06g0165161**	7,07	0,00021078	phospholipase D beta 1-like	D16	UP

**HanXRQChr17g0541251**	7,07	0,007596635	cytochrome P450 CYP72A219-like	D16	UP
**HanXRQChr02g0052981**	7,03	0,002409267	GPR107-like	D16	UP
**HanXRQChr17g0546451**	6,95	0,001538037	Late embryogenesis abundant hydroxyproline-rich glyco family,	D16	UP
**HanXRQChr15g0482311**	6,95	0,001834891	abscisic acid 8 -hydroxylase 3-like	D16	UP
**HanXRQChr16g0522771**	6,95	0,002071605	seven transmembrane domain-containing tyrosine-kinase 1-like	D16	UP
**HanXRQChr11g0322561**	6,9	0,003309288	probable indole-3-acetic acid-amido synthetase	D16	UP
**HanXRQChr04g0112871**	6,87	0,027961069	hevamine-A-like	D16	UP
**HanXRQChr08g0208461**	6,84	0,003607217	multidrug resistance	D16	UP
**HanXRQChr13g0398991**	6,83	0,000438753	ankyrin repeat and kinase domain-containing 1-like	D16	UP
**HanXRQChr07g0202141**	6,81	8,93E-05	serine threonine- kinase	D16	UP
**HanXRQChr15g0497031**	6,79	5,53E-05	ABC transporter B family member 19-like	D16	UP
**HanXRQChr11g0325481**	6,78	0,004145824	replication factor C subunit 3-like	D16	UP
**HanXRQChr15g0486991**	6,77	0,003930744	DELLA RGL1-like	D16	UP
**HanXRQChr06g0176761**	6,77	0,000275973	actin	D16	UP
**HanXRQChr06g0176161**	6,76	0,012457053	binding ,	D16	UP
**HanXRQChr12g0367481**	6,76	0,018578533	hexaprenyldihydroxybenzoate methyltransferase,	D16	UP

			mitochondrial		
**HanXRQChr07g0186251**	6,7	0,003020764	F-box and Leucine Rich Repeat domains containing , isoform 1	D16	UP
**HanXRQChr12g0374881**	6,67	0,00601415	polyol transporter 1	D16	UP
**HanXRQChr05g0150161**	6,66	0,010354705	replication A 70 kDa DNA-binding subunit C-like	D16	UP
**HanXRQChr13g0390911**	6,66	0,006388835	branched-chain-amino-acid aminotransferase 2, chloroplastic-like	D16	UP
**HanXRQChr02g0035351**	6,64	0,001148406	multidrug resistance	D16	UP
**HanXRQChr15g0474701**	6,64	1,02E-05	annexin RJ4 isoform X1	D16	UP
**HanXRQChr03g0072771**	6,62	0,003222029	cytochrome P450 CYP72A219-like	D16	UP
**HanXRQChr10g0283391**	6,59	0,000246208	zeatin O-glucosyltransferase-like	D16	UP
**HanXRQChr10g0295611**	6,59	0,00568629	germin 2-1	D16	UP
**HanXRQChr03g0087521**	6,58	0,003233046	subtilisin-like protease SDD1	D16	UP
**HanXRQChr09g0253021**	6,58	0,028839306	probable phosphatase 2C 76	D16	UP
**HanXRQChr03g0070891**	6,54	0,000432937	probable leucine-rich repeat receptor kinase At5g49770	D16	UP
**HanXRQChr16g0522031**	6,54	9,29E-05	ripening-related 1	D16	UP
**HanXRQChr16g0525131**	6,52	0,010771148	cinnamoyl- reductase 1-like	D16	UP
**HanXRQChr10g0295601**	6,5	0,010312122	germin 2-1	D16	UP
**HanXRQChr13g0395521**	6,44	0,000723252	DELLA RGL1-like	D16	UP

**HanXRQChr10g0295631**	6,43	0,01021918	germin 2-1	D16	UP
**HanXRQChr16g0532961**	6,43	0,000264846	major allergen Pru ar 1-like	D16	UP
**HanXRQChr14g0438131**	6,42	0,001176313	tubulin alpha-3 chain-like	D16	UP
**HanXRQChr10g0295541**	6,41	3,24E-05	germin 2-1	D16	UP
**HanXRQChr05g0156201**	6,41	0,002597719	PREDICTED: uncharacterized protein LOC104427809	D16	UP
**HanXRQChr17g0555721**	6,41	0,000222538	beta-galactosidase 8-like	D16	UP
**HanXRQChr16g0522641**	6,35	0,001963464	Extra-large GTP-binding 3	D16	UP
**HanXRQChr13g0399961**	6,3	0,000185253	peroxiredoxin-2E-2, chloroplastic-like	D16	UP
**HanXRQChr08g0236851**	6,26	0,015228966	serine threonine phosphatase 2A 57 kDa regulatory subunit B beta isoform-like	D16	UP
**HanXRQChr05g0162351**	6,24	0,000206353	probable serine threonine-kinase At1g54610	D16	UP
**HanXRQChr01g0006071**	6,21	0,002654402	heat shock cognate 70 kDa 2-like	D16	UP
**HanXRQChr02g0040081**	6,2	0,016544039	cytochrome P450 81E8-like	D16	UP
**HanXRQChr09g0261371**	6,12	0,018342048	ATPase family associated with various cellular activities,	D16	UP
**HanXRQChr03g0082731**	6,09	0,014706561	pectinacetylesterase family	D16	UP
**HanXRQChr02g0055931**	6,07	0,001148406	NAC domain-containing 2-like	D16	UP
**HanXRQChr10g0316841**	6,06	0,004715827	heat shock 70	D16	UP

**HanXRQChr16g0500761**	6,06	0,00036514	probable 2-oxoglutarate-dependent dioxygenase AOP1	D16	UP
**HanXRQChr17g0546621**	6,04	0,039257548	UDP-glycosyltransferase 83A1-like	D16	UP
**HanXRQChr16g0522041**	6,03	0,00116632	ripening-related 1	D16	UP
**HanXRQChr13g0415471**	6,01	1,56E-05	3-oxoacyl-[acyl-carrier-] synthase I, chloroplastic-like	D16	UP
**HanXRQChr11g0336021**	5,96	0,000196435	4-coumarate-- ligase-like 5	D16	UP
**HanXRQChr04g0111321**	5,96	0,013085911	exocyst complex component EXO84B-like	D16	UP
**HanXRQChr17g0566351**	5,96	0,001545216	PLANT CADMIUM RESISTANCE 2-like	D16	UP
**HanXRQChr15g0482331**	5,95	0,003395021	cytochrome P450 family	D16	UP
**HanXRQChr01g0006081**	5,85	2,44E-05	heat shock cognate 70 kDa 2	D16	UP
**HanXRQChr17g0555501**	5,85	0,001380642	anthocyanin acyltransferase	D16	UP
**HanXRQChr10g0291041**	5,83	0,000337269	COBRA 1	D16	UP
**HanXRQChr17g0561601**	5,83	0,015492839	seven transmembrane domain-containing tyrosine-kinase 1-like	D16	UP
**HanXRQChr08g0222171**	5,81	0,003484043	cytochrome P450 704C1-like	D16	UP
**HanXRQChr02g0042141**	5,71	0,038840607	jacalin-related lectin 3-like	D16	UP
**HanXRQChr09g0252491**	5,7	0,007596635	ferric reduction oxidase 2-like	D16	UP

**HanXRQChr17g0553141**	5,69	6,03E-05	palmitoyl-acyl carrier thioesterase, chloroplastic-like	D16	UP
**HanXRQChr03g0083931**	5,69	0,01722459	PREDICTED: uncharacterized protein LOC105140517	D16	UP
**HanXRQChr13g0393971**	5,63	0,003274279	scarecrow 3	D16	UP
**HanXRQChr09g0269291**	5,62	0,000826009	disco-interacting 2 homolog B-A-like	D16	UP
**HanXRQChr09g0250841**	5,59	0,022970249	L-type lectin-domain containing receptor kinase -like	D16	UP
**HanXRQChr16g0516831**	5,58	0,006404605	RPM1-interacting 4 isoform X2	D16	UP
**HanXRQChr01g0030791**	5,55	0,005098256	SPX domain-containing 1-like	D16	UP
**HanXRQChr04g0119621**	5,53	0,003256073	probable leucine-rich repeat receptor kinase At5g49770	D16	UP
**HanXRQChr08g0224621**	5,52	6,53E-05	cytochrome P450 CYP736A12-like	D16	UP
**HanXRQChr13g0409771**	5,51	0,031441951	beta-amyrin 28-oxidase-like	D16	UP
**HanXRQChr05g0157351**	5,46	0,016871983	unnamed protein product	D16	UP
**HanXRQChr09g0275621**	5,45	0,006144942	clavaminate synthase At3g21360	D16	UP
**HanXRQChr17g0557161**	5,44	0,01192246	ABC transporter G family member 35-like	D16	UP
**HanXRQChr10g0307321**	5,42	0,002597719	DELLA RGL1-like	D16	UP
**HanXRQChr14g0448601**	5,39	0,000254012	ethylene-responsive transcription factor RAP2-	D16	UP

			2-like		
**HanXRQChr07g0204551**	5,35	0,003479686	very-long-chain enoyl-reductase-like	D16	UP
**HanXRQChr03g0089731**	5,34	0,001116416	zinc finger 281-like	D16	UP
**HanXRQChr15g0476021**	5,32	0,000327197	clavaminate synthase At3g21360	D16	UP
**HanXRQChr17g0554241**	5,3	0,009018625	anthocyanin acyltransferase	D16	UP
**HanXRQChr17g0565271**	5,3	0,013258897	F-box CPR30-like	D16	UP
**HanXRQChr17g0570651**	5,21	0,000602629	L-type lectin-domain containing receptor kinase -like	D16	UP
**HanXRQChr17g0534891**	5,21	0,002568282	dynamin-related 5A	D16	UP
**HanXRQChr13g0410761**	5,19	0,005143931	fructan 1-exohydrolase	D16	UP
**HanXRQChr15g0484141**	5,12	0,028733471	vacuolar iron transporter homolog 2-like	D16	UP
**HanXRQChr10g0309041**	5,1	0,005643741	probable LRR receptor-like serine threonine- kinase At1g56140	D16	UP
**HanXRQChr14g0427931**	5,03	0,000262549	pyrophosphate--fructose 6-phosphate 1-phosphotransferase subunit alpha	D16	UP
**HanXRQChr02g0052781**	4,91	0,000521827	lysosomal beta glucosidase-like	D16	UP
**HanXRQChr10g0306811**	4,91	0,000375196	hypothetical protein MIMGU_mgv1a021450mg	D16	UP
**HanXRQChr10g0293971**	4,89	0,040723183	probable aldo-keto reductase 2	D16	UP

**HanXRQChr08g0208831**	4,88	0,001382781	Serine-threonine kinase,	D16	UP
**HanXRQChr10g0281681**	4,79	0,001039146	acidic mammalian chitinase-like	D16	UP
**HanXRQChr03g0075011**	4,78	0,001178147	transmembrane emp24 domain-containing p24beta2-like	D16	UP
**HanXRQChr12g0376571**	4,77	0,000628202	2-alkenal reductase (NADP(+)-dependent)-like	D16	UP
**HanXRQChr04g0125541**	4,74	0,000669982	probable S-acyltransferase 4	D16	UP
**HanXRQChr09g0271641**	4,73	0,036830092	formin 4	D16	UP
**HanXRQChr10g0285741**	4,68	0,003418062	ATP binding cassette subfamily B4 isoform 2	D16	UP
**HanXRQChr11g0348641**	4,68	0,002559267	ankyrin repeat and kinase domain-containing 1-like	D16	UP
**HanXRQChr13g0400761**	4,65	0,000746027	abscisic-aldehyde oxidase-like	D16	UP
**HanXRQChr08g0208451**	4,65	0,00039618	Os02g0189700	D16	UP
**HanXRQChr13g0407671**	4,63	0,001181278	multidrug resistance	D16	UP
**HanXRQChr09g0272491**	4,63	0,000770199	multidrug resistance	D16	UP
**HanXRQChr15g0464481**	4,62	0,020069792	ATP binding cassette subfamily B4 isoform 2	D16	UP
**HanXRQChr10g0295511**	4,62	0,000169098	germin 2-1	D16	UP
**HanXRQChr11g0320971**	4,61	0,000643583	sulfate transporter -like	D16	UP
**HanXRQChr01g0026341**	4,53	0,000141522	lysosomal beta glucosidase-like	D16	UP
**HanXRQChr01g0004391**	4,51	0,034592899	dammarenediol II synthase-like	D16	UP

**HanXRQChr10g0292491**	4,5	0,003513439	cytochrome P450 93A3-like	D16	UP
**HanXRQChr12g0382471**	4,5	0,01732083	receptor kinase FERONIA	D16	UP
**HanXRQChr15g0472231**	4,49	2,04E-05	NRT1 PTR FAMILY -like	D16	UP
**HanXRQChr02g0048491**	4,49	0,032662184	DELLA RGL1-like	D16	UP
**HanXRQChr02g0037181**	4,46	0,032213491	probable inactive receptor kinase At2g26730	D16	UP
**HanXRQChr01g0031131**	4,43	0,046987421	cellulose synthase A catalytic subunit 1 [UDP-forming]	D16	UP
**HanXRQChr14g0434241**	4,42	0,041036941	probable calcium-binding CML44	D16	UP
**HanXRQChr15g0464441**	4,41	7,97E-06	ABC transporter B family member 4-like	D16	UP
**HanXRQChr12g0379281**	4,39	0,000573672	DUF827 family	D16	UP
**HanXRQChr07g0202551**	4,38	0,02911138	Disease resistance family LRR family	D16	UP
**HanXRQChr03g0080301**	4,38	0,001464462	LEM3 (ligand-effect modulator 3) family CDC50 family isoform 1	D16	UP
**HanXRQChr15g0489211**	4,33	0,046810388	receptor kinase FERONIA	D16	UP
**HanXRQChr15g0489841**	4,3	0,000150054	probable serine threonine-kinase At1g54610	D16	UP
**HanXRQChr02g0052241**	4,29	0,002239199	PREDICTED: uncharacterized protein LOC101246082	D16	UP
**HanXRQChr10g0281501**	4,28	0,000155649	heat shock cognate 70 kDa 2-like	D16	UP

**HanXRQChr13g0400591**	4,25	0,003059273	UDP-glycosyltransferase 73C3-like	D16	UP
**HanXRQChr08g0210941**	4,24	0,002313115	extracellular ribonuclease LE-like	D16	UP
**HanXRQChr03g0079881**	4,23	0,021411825	mitochondrial substrate carrier family P	D16	UP
**HanXRQChr17g0546581**	4,23	0,00246093	UDP-glycosyltransferase 83A1-like	D16	UP
**HanXRQChr09g0244221**	4,22	0,00039618	vacuolar-sorting receptor 1-like	D16	UP
**HanXRQChr12g0372661**	4,2	0,048756693	2-oxoglutarate-dependent dioxygenase DAO-like	D16	UP
**HanXRQChr07g0206991**	4,17	0,004063265	Cytosolic beta-glucosidase	D16	UP
**HanXRQChr03g0072751**	4,16	0,021714255	cytochrome P450 family 72	D16	UP
**HanXRQChr15g0474511**	4,15	0,003203279	probable inactive receptor kinase At2g26730	D16	UP
**HanXRQChr05g0157371**	4,11	0,001156687	cytochrome P450 CYP82D47-like	D16	UP
**HanXRQChr06g0170641**	4,07	0,002398275	triacylglycerol lipase,	D16	UP
**HanXRQChr09g0254241**	4,05	0,002177778	SCARECROW-like	D16	UP
**HanXRQChr17g0554261**	4,05	0,013067949	anthocyanin acyltransferase	D16	UP
**HanXRQChr09g0268021**	4,04	0,003985062	serine carboxypeptidase II-3-like	D16	UP
**HanXRQChr10g0281011**	4,03	0,013101006	hyoscyamine 6-dioxygenase-like	D16	UP
**HanXRQChr10g0316541**	4	0,006092209	PREDICTED: uncharacterized protein	D16	UP

			LOC105975005		
**HanXRQChr15g0473771**	3,98	0,002448047	salicylic acid-binding 2-like	D16	UP
**HanXRQChr13g0395531**	3,94	0,000643583	DELLA RGL1-like	D16	UP
**HanXRQChr17g0561331**	3,92	0,035679955	tropinone reductase homolog	D16	UP
**HanXRQChr08g0237281**	3,9	0,00085136	NRT1 PTR FAMILY -like	D16	UP
**HanXRQChr02g0055621**	3,89	9,25E-05	serine carboxypeptidase-like	D16	UP
**HanXRQChr07g0199371**	3,87	0,026038754	NAC domain-containing 94	D16	UP
**HanXRQChr03g0072231**	3,87	0,000137552	serine carboxypeptidase II-3-like	D16	UP
**HanXRQChr14g0428571**	3,85	0,021808554	F-box RNI FBD-like domain	D16	UP
**HanXRQChr14g0447381**	3,84	0,009497417	wall-associated receptor kinase-like 1	D16	UP
**HanXRQChr09g0254161**	3,82	0,03752259	retrotransposon like	D16	UP
**HanXRQChr17g0562461**	3,78	0,002239199	probable calcium-binding CML23	D16	UP
**HanXRQChr10g0299221**	3,75	0,004373065	receptor kinase At4g00960	D16	UP
**HanXRQChr04g0096191**	3,71	0,00013151	NRT1 PTR FAMILY	D16	UP
**HanXRQChr14g0430021**	3,68	0,003874138	heparan-alpha-glucosaminide N-acetyltransferase-like	D16	UP
**HanXRQChr03g0085721**	3,67	4,81E-05	S-norcoclaurine synthase 1-like	D16	UP
**HanXRQChr16g0509901**	3,67	0,046191841	---NA---	D16	UP
**HanXRQChr05g0131961**	3,67	0,012677575	NRT1 PTR FAMILY -like	D16	UP

**HanXRQChr11g0330581**	3,65	0,016591063	2-oxoglutarate and Fe(II)-dependent oxygenase superfamily	D16	UP
**HanXRQChr15g0468811**	3,64	0,019976302	cellulose synthase G3	D16	UP
**HanXRQChr09g0243551**	3,62	0,002568282	deacetylvindoline O-acetyltransferase-like	D16	UP
**HanXRQChr07g0191631**	3,56	0,002121142	probable mitochondrial chaperone bcs1	D16	UP
**HanXRQChr08g0233781**	3,55	0,006388835	---NA---	D16	UP
**HanXRQChr09g0264761**	3,54	0,006588601	scarecrow 3	D16	UP
**HanXRQChr11g0326171**	3,54	0,00667619	beta-galactosidase 16-like	D16	UP
**HanXRQChr10g0287961**	3,52	0,000312981	wound-induced 1	D16	UP
**HanXRQChr14g0458661**	3,5	0,007359099	UDP-glycosyltransferase 76G1	D16	UP
**HanXRQChr09g0242821**	3,48	0,007280421	uncharacterized LOC102600333	D16	UP
**HanXRQChr16g0500771**	3,47	0,000435844	probable 2-oxoglutarate-dependent dioxygenase AOP1	D16	UP
**HanXRQChr05g0142231**	3,47	0,000955513	probable LRR receptor-like serine threonine- kinase At1g56140	D16	UP
**HanXRQChr07g0190311**	3,46	0,000203387	cytochrome P450 CYP72A219-like	D16	UP
**HanXRQChr12g0363041**	3,43	0,00246093	14-3-3 GF14 iota	D16	UP
**HanXRQChr04g0102111**	3,43	0,01820974	7-deoxyloganetin glucosyltransferase-like	D16	UP

**HanXRQChr12g0372301**	3,41	0,004840468	Ankyrin repeat	D16	UP
**HanXRQChr13g0391111**	3,41	0,0207414	transcription factor MYB12	D16	UP
**HanXRQChr02g0036801**	3,4	0,035960608	---NA---	D16	UP
**HanXRQChr13g0416521**	3,39	0,019976302	nuclear transcription factor Y subunit C-2-like	D16	UP
**HanXRQChr02g0045921**	3,38	0,006878649	probable rhamnogalacturonate lyase B isoform X1	D16	UP
**HanXRQChr03g0072761**	3,31	0,005098256	cytochrome P450 CYP72A219-like	D16	UP
**HanXRQChr15g0464431**	3,29	0,000995023	ABC transporter B family member 11-like	D16	UP
**HanXRQChr08g0234891**	3,28	0,00236159	probable methyltransferase PMT2	D16	UP
**HanXRQChr06g0185291**	3,22	0,031282442	MLO homolog 1-like	D16	UP
**HanXRQChr11g0323461**	3,21	0,044585988	RING-H2 finger ATL11-like	D16	UP
**HanXRQChr03g0090191**	3,21	0,007700141	SHORT-ROOT-like	D16	UP
**HanXRQChr05g0150361**	3,19	0,040174616	---NA---	D16	UP
**HanXRQChr11g0338341**	3,17	0,018515995	taxadiene 5-alpha hydroxylase-like	D16	UP
**HanXRQChr03g0063761**	3,11	0,001982439	endochitinase PR4-like	D16	UP
**HanXRQChr04g0094461**	3,11	0,0464217	ABC transporter B family member 19-like	D16	UP
**HanXRQChr04g0120371**	3,11	0,013527439	cytochrome P450 78A3p family	D16	UP
**HanXRQChr16g0500751**	3,09	0,046987421	probable 2-oxoglutarate-	D16	UP

			dependent dioxygenase AOP1		
**HanXRQChr14g0430931**	3,08	0,037371077	receptor kinase FERONIA	D16	UP
**HanXRQChr15g0486091**	3,06	0,004063265	PTI1-like tyrosine- kinase At3g15890	D16	UP
**HanXRQChr15g0492151**	3,06	0,016544039	probable aquaporin TIP3-2	D16	UP
**HanXRQChr07g0194211**	3,03	0,000770199	kinase G11A-like	D16	UP
**HanXRQChr14g0462551**	3,01	0,035484354	heat shock 83	D16	UP
**HanXRQChr04g0112301**	2,99	0,003265043	NRT1 PTR FAMILY -like	D16	UP
**HanXRQChr11g0320141**	2,97	0,000244623	PREDICTED: uncharacterized protein LOC104231606 isoform X2	D16	UP
**HanXRQChr09g0264461**	2,95	0,000807243	probable indole-3-acetic acid-amido synthetase	D16	UP
**HanXRQChr07g0190101**	2,95	0,012141027	serine-threonine kinase, plant-type,	D16	UP
**HanXRQChr15g0471391**	2,94	0,0128524	glucose-6-phosphate phosphate translocator 2, chloroplastic-like	D16	UP
**HanXRQChr08g0218881**	2,94	0,001065451	auxin response factor 18-like	D16	UP
**HanXRQChr05g0141911**	2,92	0,026096891	scarecrow 21	D16	UP
**HanXRQChr03g0089421**	2,92	0,024990098	glyoxysomal fatty acid beta-oxidation multifunctional MFP-a	D16	UP
**HanXRQChr01g0009951**	2,91	0,001674663	exocyst complex component EXO70A1-like	D16	UP

**HanXRQChr02g0056151**	2,89	0,017831964	pheromone receptor	D16	UP
**HanXRQChr02g0052341**	2,89	0,010312122	29 kDa ribonucleo A, chloroplastic-like	D16	UP
**HanXRQChr03g0068281**	2,89	0,0122199	serine carboxypeptidase II-3-like	D16	UP
**HanXRQChr09g0266411**	2,87	0,010354705	7-deoxyloganetin glucosyltransferase-like	D16	UP
**HanXRQChr11g0331311**	2,87	0,000337231	Salicylate O-methyltransferase	D16	UP
**HanXRQChr13g0394631**	2,86	0,00577874	PREDICTED: uncharacterized protein LOC104600249	D16	UP
**HanXRQChr12g0377021**	2,85	0,000770199	NADPH-dependent 1-acyldihydroxyacetone phosphate reductase-like	D16	UP
**HanXRQChr14g0440871**	2,84	0,017485508	glutamate receptor -like	D16	UP
**HanXRQChr11g0335651**	2,84	0,003213842	glyceraldehyde-3-phosphate dehydrogenase GAPCP2, chloroplastic	D16	UP
**HanXRQChr11g0337971**	2,84	9,18E-05	UDP-glycosyltransferase 76E2-like	D16	UP
**HanXRQChr13g0398681**	2,83	0,003436858	calcium-dependent kinase 26-like	D16	UP
**HanXRQChr11g0329431**	2,82	0,000418696	PREDICTED: uncharacterized protein LOC103329132	D16	UP
**HanXRQChr11g0330961**	2,79	0,001792271	beta-hexosaminidase 2	D16	UP
**HanXRQChr14g0461901**	2,77	0,000321956	cytochrome P450 704C1-like	D16	UP

**HanXRQChr08g0211861**	2,77	0,000995023	beta-glucosidase 40-like	D16	UP
**HanXRQChr16g0527131**	2,77	0,004289377	cytochrome P450 89A2-like	D16	UP
**HanXRQChr07g0203151**	2,71	0,001605688	U-box domain-containing 13-like	D16	UP
**HanXRQChr01g0029651**	2,71	0,01833235	NRT1 PTR FAMILY -like isoform X1	D16	UP
**HanXRQChr12g0357381**	2,71	0,00292238	PLASTID MOVEMENT IMPAIRED 2	D16	UP
**HanXRQChr04g0113701**	2,7	0,007387594	cytochrome P450 94A2-like	D16	UP
**HanXRQChr14g0449451**	2,69	0,01998287	type I inositol 1,4,5-trisphosphate 5-phosphatase CVP2-like isoform X1	D16	UP
**HanXRQChr14g0452921**	2,69	0,000391561	E3 ubiquitin- ligase LIN-1	D16	UP
**HanXRQChr03g0085511**	2,68	0,019637004	---NA---	D16	UP
**HanXRQChr13g0389241**	2,66	0,027961069	early nodulin 2	D16	UP
**HanXRQChr15g0464421**	2,66	0,000968517	ABC transporter B family member 11-like	D16	UP
**HanXRQChr13g0407961**	2,66	0,011543819	ABC-1 domain	D16	UP
**HanXRQChr14g0435281**	2,63	0,000607502	protochlorophyllide-dependent translocon component 52, chloroplastic-like	D16	UP
**HanXRQChr01g0020731**	2,62	0,003865997	probable purine permease 10	D16	UP
**HanXRQChr03g0072311**	2,61	0,000558578	serine carboxypeptidase II-3-like	D16	UP
**HanXRQChr06g0172981**	2,61	0,012731757	transmembrane ,	D16	UP

**HanXRQChr15g0471211**	2,56	0,043584542	cytochrome P450 716B1-like	D16	UP
**HanXRQChr05g0141441**	2,56	0,000621167	acidic mammalian chitinase-like	D16	UP
**HanXRQChr03g0069731**	2,56	0,006878649	receptor kinase FERONIA	D16	UP
**HanXRQChr11g0330071**	2,55	0,000834239	zinc finger NUTCRACKER-like	D16	UP
**HanXRQChr05g0137931**	2,54	0,033764103	plant F25P12-18	D16	UP
**HanXRQChr07g0187961**	2,54	0,007322871	Nicotinate phosphoribosyltransferase	D16	UP
**HanXRQChr06g0173731**	2,52	0,038941927	epoxide hydrolase	D16	UP
**HanXRQChr09g0254101**	2,52	0,010700404	receptor-like cytosolic serine threonine- kinase RBK1	D16	UP
**HanXRQChr02g0052021**	2,51	0,017827403	DUF3411 domain	D16	UP
**HanXRQChr07g0206831**	2,51	0,02365501	MATE efflux family LAL5-like	D16	UP
**HanXRQChr09g0265311**	2,51	0,006100502	NAC domain-containing ,	D16	UP
**HanXRQChr06g0171461**	2,51	0,023655283	probable metal-nicotianamine transporter YSL7	D16	UP
**HanXRQChr05g0150891**	2,5	0,039432459	alpha-amylase	D16	UP
**HanXRQChr14g0435291**	2,49	0,000643583	protochlorophyllide-dependent translocon component 52, chloroplastic-like	D16	UP
**HanXRQChr08g0218261**	2,46	0,036827165	4-coumarate-- ligase-like 5	D16	UP

**HanXRQChr03g0060291**	2,44	0,045623949	diacylglycerol O-acyltransferase 1	D16	UP
**HanXRQChr17g0557151**	2,41	0,010543228	ABC transporter G family member 29-like	D16	UP
**HanXRQChr12g0373981**	2,41	0,027961069	F-box SNE-like	D16	UP
**HanXRQChr12g0375131**	2,38	0,018515995	PREDICTED: uncharacterized protein LOC104113151	D16	UP
**HanXRQChr01g0026951**	2,38	0,026768338	benzyl alcohol O-benzoyltransferase	D16	UP
**HanXRQChr10g0308851**	2,38	0,021411825	CHLOROPLAST IMPORT APPARATUS 2-like	D16	UP
**HanXRQChr05g0136401**	2,36	0,005955684	BRCT domain-containing DNA repair	D16	UP
**HanXRQChr13g0407681**	2,35	0,021835452	TBC1 domain family member 15-like	D16	UP
**HanXRQChr09g0255531**	2,34	0,000498045	glycerol-3-phosphate dehydrogenase [NAD(+)]	D16	UP
**HanXRQChr12g0355121**	2,34	0,04797473	mitochondrial import inner membrane translocase subunit TIM17-2-like	D16	UP
**HanXRQChr14g0447471**	2,33	0,032176504	actin, partial	D16	UP
**HanXRQChr15g0478361**	2,33	0,00442431	probable transcription factor 21	D16	UP
**HanXRQChr01g0009621**	2,33	0,044652985	Myb-like HTH transcriptional regulator family , isoform 1	D16	UP
**HanXRQChr09g0254091**	2,33	0,023920637	serine threonine- kinase At5g01020-like	D16	UP

**HanXRQChr15g0465221**	2,32	0,000723252	---NA---	D16	UP
**HanXRQChr04g0094701**	2,3	0,011760711	epoxide hydrolase 4	D16	UP
**HanXRQChr02g0036791**	2,3	0,00507192	UDP-glycosyltransferase 85A5-like	D16	UP
**HanXRQChr14g0458641**	2,3	0,033639769	UDP-glycosyltransferase 76G1	D16	UP
**HanXRQChr08g0215061**	2,22	0,000435934	Thioredoxin superfamily	D16	UP
**HanXRQChr16g0517481**	2,19	0,01021918	Vesicle-associated 4-2	D16	UP
**HanXRQChr04g0120241**	2,16	0,004675619	triacylglycerol lipase,	D16	UP
**HanXRQChr17g0570641**	2,15	0,044652985	L-type lectin-domain containing receptor kinase -like	D16	UP
**HanXRQChr01g0008761**	2,14	0,015882443	villin-2	D16	UP
**HanXRQChr11g0333481**	2,14	0,000318985	Cytochrome P450 71D10,	D16	UP
**HanXRQChr15g0472811**	2,12	0,019845405	alpha beta hydrolase domain-containing 17B-like	D16	UP
**HanXRQChr10g0279991**	2,11	0,006168206	vesicle-associated membrane 726	D16	UP
**HanXRQChr03g0092311**	2,11	0,046914034	cellulose synthase G2	D16	UP
**HanXRQChr04g0103891**	2,08	0,0074508	FAM179B-like isoform X2	D16	UP
**HanXRQChr06g0164031**	2,08	0,002409267	CSC1 At4g02900	D16	UP
**HanXRQChr10g0318701**	2,07	0,003395021	thioredoxin-dependent peroxidase	D16	UP
**HanXRQChr08g0227311**	2,07	0,019275435	laccase-1-like	D16	UP
**HanXRQChr17g0536311**	2,06	0,039478398	phospholipid-transporting ATPase 5	D16	UP

**HanXRQChr05g0143521**	2,06	0,008254636	synaptotagmin-5-like	D16	UP
**HanXRQChr05g0162211**	2,06	0,046820972	phosphoglycerate mutase-like	D16	UP
**HanXRQChr11g0341411**	2,05	0,01129416	probable aldo-keto reductase 2	D16	UP
**HanXRQChr14g0444401**	2	0,008064068	mannitol dehydrogenase	D16	UP
**HanXRQChr13g0406771**	1,99	0,001124479	actin	D16	UP
**HanXRQChr07g0200361**	1,93	0,001307307	COBRA-like isoform X1	D16	UP
**HanXRQChr04g0114361**	1,93	0,025394935	pyruvate dehydrogenase E1 component subunit beta-3, chloroplastic-like	D16	UP
**HanXRQChr03g0091831**	1,92	0,000347903	anthocyanidin 3-O-glucosyltransferase 2-like	D16	UP
**HanXRQChr02g0053981**	1,92	0,042224136	Glycosyl hydrolases family 31 isoform 1	D16	UP
**HanXRQChr08g0225831**	1,92	0,002983521	UNC93 1	D16	UP
**HanXRQChr05g0142101**	1,9	0,02650666	G-type lectin S-receptor-like serine threonine- kinase	D16	UP
**HanXRQChr01g0013791**	1,89	0,01340083	copalyl diphosphate synthase	D16	UP
**HanXRQChr04g0102781**	1,89	0,003551813	exocyst complex component EXO70A1-like	D16	UP
**HanXRQChr03g0066951**	1,86	0,009903304	beta-hydroxyacyl-ACP dehydratase	D16	UP
**HanXRQChr08g0223051**	1,84	0,019976302	Ras-related RABD2c	D16	UP
**HanXRQChr13g0413291**	1,84	0,024270349	monogalactosyldiacylglycerol synthase, chloroplastic	D16	UP

**HanXRQChr03g0087781**	1,84	0,04938835	metalloendo ase 1-like	D16	UP
**HanXRQChr17g0557101**	1,83	0,019845405	Extended synaptotagmin-2	D16	UP
**HanXRQChr09g0242241**	1,83	0,001762845	2-alkenal reductase (NADP(+)-dependent)-like	D16	UP
**HanXRQChr09g0247351**	1,81	0,035293568	auxin response factor 16-like	D16	UP
**HanXRQChr14g0449531**	1,79	0,003177084	probable indole-3-acetic acid-amido synthetase	D16	UP
**HanXRQChr12g0358551**	1,76	0,025766341	beta-amyrin 28-oxidase-like	D16	UP
**HanXRQChr02g0044471**	1,75	0,0464217	heat shock cognate 70 kDa 2-like	D16	UP
**HanXRQChr09g0276651**	1,74	0,006645721	aminopeptidase M1-like	D16	UP
**HanXRQChr08g0221671**	1,74	0,001148406	methylesterase 17	D16	UP
**HanXRQChr09g0274771**	1,73	0,043584542	clustered mitochondria homolog	D16	UP
**HanXRQChr07g0189601**	1,72	0,002248512	SRG1-like	D16	UP
**HanXRQChr08g0221471**	1,71	0,013354374	7-deoxyloganetic acid glucosyltransferase-like	D16	UP
**HanXRQChr05g0151771**	1,69	0,01833235	auxilin 1	D16	UP
**HanXRQChr09g0276941**	1,69	0,004084077	nodulation-signaling pathway 1 -like	D16	UP
**HanXRQChr14g0462681**	1,69	0,025829214	plastidial pyruvate kinase 2	D16	UP
**HanXRQChr09g0239381**	1,69	0,02877708	E3 ubiquitin- ligase LIN-1	D16	UP
**HanXRQChr06g0184771**	1,67	0,004395125	hexokinase-2, chloroplastic	D16	UP
**HanXRQChr13g0400631**	1,66	0,038046056	UDP-glycosyltransferase 73C3-like	D16	UP

**HanXRQChr09g0241851**	1,66	0,037382423	Octicosapeptide Phox Bem1p domain-containing tetratricopeptide repeat-containing	D16	UP
**HanXRQChr07g0192541**	1,66	0,010771148	UPF0548 At2g17695	D16	UP
**HanXRQChr12g0372291**	1,64	0,003365675	4-coumarate-- ligase-like 9	D16	UP
**HanXRQChr04g0121211**	1,62	0,005381239	bifunctional L-3-cyanoalanine synthase cysteine synthase 1, mitochondrial	D16	UP
**HanXRQChr04g0116711**	1,6	0,040555601	NRT1 PTR FAMILY -like	D16	UP
**HanXRQChr15g0485501**	1,59	0,011199229	UDP-glycosyltransferase 73C3-like	D16	UP
**HanXRQChr04g0108311**	1,56	0,042681201	quinone-oxidoreductase homolog, chloroplastic	D16	UP
**HanXRQChr01g0027851**	1,56	0,01820974	UDP-glycosyltransferase 73C3-like	D16	UP
**HanXRQChr02g0049441**	1,56	0,016441202	3-oxoacyl-[acyl-carrier-] synthase 3 A, chloroplastic	D16	UP
**HanXRQChr10g0299371**	1,54	0,01132653	dihydrolipoyl dehydrogenase 2, chloroplastic-like	D16	UP
**HanXRQChr16g0510881**	1,54	0,020143657	transmembrane ,	D16	UP
**HanXRQChr15g0483951**	1,53	0,036520087	lysosomal alpha-mannosidase	D16	UP
**HanXRQChr14g0442541**	1,51	0,008064068	Transducin WD40 repeat-like superfamily isoform 1	D16	UP
**HanXRQChr10g0283961**	1,51	0,02252704	potassium transporter 5-like	D16	UP

**HanXRQChr08g0208171**	1,48	0,022696036	UDP-galactose:fucoside alpha-3-galactosyltransferase	D16	UP
**HanXRQChr12g0367211**	1,47	0,031282442	E3 ubiquitin- ligase LIN-1 isoform X1	D16	UP
**HanXRQChr17g0549151**	1,46	0,042812114	flavonoid 3 -monooxygenase-like	D16	UP
**HanXRQChr10g0284751**	1,46	0,012636993	gibberellin 2-beta-dioxygenase 2	D16	UP
**HanXRQChr10g0314191**	1,44	0,018327739	heparanase 3	D16	UP
**HanXRQChr00c0143g0572051**	1,44	0,025634964	oligopeptide transporter 4-like	D16	UP
**HanXRQChr01g0025251**	1,43	0,026211496	secoisolariciresinol dehydrogenase-like	D16	UP
**HanXRQChr09g0255681**	1,41	0,019435473	---NA---	D16	UP
**HanXRQChr13g0408101**	1,41	0,027096618	O-linked-mannose beta-1,4-N-acetylglucosaminyltransferase 2-like	D16	UP
**HanXRQChr04g0101951**	1,4	0,040047678	UDP-glycosyltransferase 85A2-like	D16	UP
**HanXRQChr02g0042491**	1,39	0,043584542	biotin carboxyl carrier of acetyl- carboxylase 1, chloroplastic-like	D16	UP
**HanXRQChr03g0089451**	1,35	0,046987421	peroxisomal fatty acid beta-oxidation multifunctional MFP2	D16	UP
**HanXRQChr14g0431481**	1,29	0,043584542	scarecrow 32	D16	UP
**HanXRQChr06g0168891**	-9,94	0,041148908	chromatin modification-related EAF1-like isoform	D16	DOWN

			X2		
**HanXRQChr04g0094591**	-4,42	0,022985067	probable (S)-N-methylcoclaurine 3 -hydroxylase isozyme 2	D16	DOWN
**HanXRQChr01g0000551**	-3,62	0,021957219	serine threonine- kinase 2 19-like	D16	DOWN
**HanXRQChr14g0456181**	-3,01	0,003903255	non-symbiotic hemoglobin 2	D16	DOWN
**HanXRQChr08g0210821**	-2,68	0,024893196	Alpha-expansin 1 precursor,	D16	DOWN
**HanXRQChr06g0164151**	-2,44	0,025079606	vacuolar-processing enzyme	D16	DOWN
**HanXRQChr16g0498441**	-2,15	0,006121576	bidirectional sugar transporter SWEET12-like	D16	DOWN
**HanXRQChr09g0273571**	-1,85	0,00943188	bidirectional sugar transporter SWEET12-like	D16	DOWN
**HanXRQChr09g0273731**	-1,82	0,006166169	bidirectional sugar transporter SWEET12-like	D16	DOWN
**HanXRQChr02g0055191**	-1,51	0,040110544	pathogen-related -like	D16	DOWN
**HanXRQChr03g0082651**	-1,49	0,041854636	probable receptor kinase At2g23200	D16	DOWN
**HanXRQChr08g0226521**	-1,36	0,046914034	peroxidase 24-like	D16	DOWN
**HanXRQChr06g0173671**	-1,3	0,036032791	ethylene-responsive transcription factor RAP2-3	D16	DOWN
**HanXRQChr03g0087431**	8,46	0,026760711	mavicyanin-like	D4	UP
**HanXRQChr12g0354981**	7,78	0,009498541	Rhicadhesin receptor precursor,	D4	UP
**HanXRQChr15g0472261**	7,32	0,0019159	NRT1 PTR FAMILY -like	D4	UP
**HanXRQChr03g0087411**	7,08	0,005032243	mavicyanin-like	D4	UP

**HanXRQChr01g0024241**	6,58	0,024319219	Subtilisin-like protease	D4	UP
**HanXRQChr03g0072271**	6,07	0,024319219	serine carboxypeptidase II-3-like	D4	UP
**HanXRQChr11g0350641**	5,63	0,010886877	UDP-glycosyltransferase 73C3-like	D4	UP
**HanXRQChr15g0490031**	5,43	0,031343524	nodulin-26-like	D4	UP
**HanXRQChr03g0087471**	5,32	0,0019159	Subtilisin-like protease SDD1	D4	UP
**HanXRQChr15g0488781**	5,11	0,048854431	UDP-glycosyltransferase 83A1-like	D4	UP
**HanXRQChr05g0159761**	5,01	0,048161659	chitinase 2-like	D4	UP
**HanXRQChr04g0127841**	4,88	0,007280114	ammonium transporter 3 member 1-like	D4	UP
**HanXRQChr06g0174451**	4,76	0,014611041	lysM domain receptor-like kinase 3	D4	UP
**HanXRQChr09g0257681**	4,32	0,01562671	germin 2-1	D4	UP
**HanXRQChr10g0280991**	4,32	0,038602554	inorganic phosphate transporter 1-4-like isoform X2	D4	UP
**HanXRQChr10g0295581**	4,26	0,01562671	germin 2-1	D4	UP
**HanXRQChr13g0419211**	4,04	0,008723509	subtilisin-like protease	D4	UP
**HanXRQChr03g0074111**	4	0,01455193	unnamed protein product	D4	UP
**HanXRQChr10g0295541**	3,75	0,031869431	germin 2-1	D4	UP

Supplementary Tab. 2 Gene ID, description and primers used in qRT-PCR analysis.

**Genome ID**	**DESCRIPTION**	**PRIMERS**
**HanXRQChr10g0283961**	Potassium transporter 5-like	FORWARD
		CATAGAAGTCCCAAGCATCGCAA
		REVERSE
		CGTAACACAATATCATCAAAGGTCA
**HanXRQChr11g0331311**	Salycilate o-methyltransferase	FORWARD
		CCACCACATACTACTTCCTCAG
		REVERSE
		ACAAGTCCTCCAAATGTATTTCAC
**HanXRQChr08g0237281**	protein nrt1 ptr family-like	FORWARD
		GCCGATATTGATGGAGAAGTAG
		REVERSE
		CTCTAGGAACAGGAGGTATTAAAC
**HanXRQChr03g0087521**	Subtilisin-like protease	FORWARD
		GCGACTCCTTCTTTGTAACCA
		REVERSE
		TGCGATGATTCTACTTAACCAAGCCT
**HanXRQChr17g0562461**	probable calcium protein cml23	FORWARD
		AGCACAACAAGAGATACGAGTAG
		REVERSE
		TAAGTCAATGATGTCCATAGGTAT

**HanXRQChr05g0138451**	e3 ubiquitin-protein ligase bre1-like 1	FORWARD
		CGCCATTGACCTGAAAACTCCAG
		REVERSE
		GCATGTGTCCAGAAGATCATTGAA
**HanXRQChr10g0282971**	mitochondrial ubiquitin ligase activator of nfkb 1	FORWARD
		CTGCATCCTTTCCGCTACTTCTG
		REVERSE
		CCGTTAATCCCTTTGGTACACTA
